# Unmet clinical needs in women with polycystic ovary syndrome regarding fertility and obesity: a cross-sectional study from the patient’s perspective

**DOI:** 10.1007/s00404-024-07916-1

**Published:** 2025-01-25

**Authors:** Annette Bachmann, Susanna Weidlinger, Michael von Wolff, Norman Bitterlich, Thomas Karn, Julia Estermann, Marina Sourouni, Petra Stute

**Affiliations:** 1 Goethe University Frankfurt: Goethe-Universitat Frankfurt am Main, Frankfurt, Germany; 2 Faculty of medicine, University of Bern, Bern, Switzerland; 3 Freelance, statistician, Chemnitz, Germany; 4 Division of Gynaecological Endocrinology and Reproductive Medicine, University Hospital Inselspital, Bern, Switzerland; 5 Division of Gynaecological Endocrinology and Reproductive Medicine, Department of Gynaecology and Obstetrics, University Hospital Frankfurt, Frankfurt, Germany; 6 Department for Gynaecological Endocrinology and Fertility Disorders, University Hospital Heidelberg, Heidelberg, Germany

**Keywords:** Polycystic ovary syndrome, Obesity, Infertility, Gynecological care, Patient satisfaction

## Abstract

**Purpose:**

Polycystic ovary syndrome (PCOS) is a common endocrine disorder in women of reproductive age, often leading to anovulatory infertility. Obesity exacerbates the reproductive, metabolic and psychological features of PCOS, making fertility treatment and patient satisfaction difficult. Despite guidelines from the European Society of Human Reproduction and Embryology (ESHRE) emphasizing lifestyle modifications and specific treatments, there remains a significant gap in adherence to these guidelines by both healthcare providers and patients.

**Methods:**

A cross-sectional online survey was conducted from 1 January to 14 March 2021 among PCOS patients in Germany, Austria and Switzerland. A non-standardized, non-validated questionnaire covering several aspects of reproductive health was distributed via online channels. Data were analyzed using descriptive statistics, chi-squared tests, Student’s t-tests and Jonckheere-Terpstra tests, with significance set at p < 0.05.

**Results:**

Out of 2029 participants, 1902 completed the fertility questionnaire. Of these, 73.9% perceived their fertility to be impaired, with this perception being higher in obese women (80.8% vs. 67.4%, p < 0.001). The analysis focused on 564 childless women with a current desire to have children, 67.0% of whom met WHO criteria for infertility. Obese women (BMI ≥ 30 kg/m^2^) reported lower satisfaction with fertility treatment (40.9 vs. 47.8, p = 0.009) and were less likely to receive fertility treatment (56.7% vs. 75.8%, p < 0.001). Despite recommendations, only 34.1% reported lifestyle changes as part of their treatment. Letrozole, the recommended first-line treatment, was underused (14.6%) and clomiphene citrate was more commonly prescribed (35.4%). Obese women reported fewer current pregnancies (4% vs. 13.9%, p < 0.001) and were more likely to be infertile for more than one year (77.0% vs. 53.0%, p < 0.001). They also expressed a greater desire for possibilities to ask more questions about PCOS and fertility and to undergo more infertility tests. (56.1% vs. 45.3%, p = 0.013; 69.4% vs. 59.8%, p = 0.020).

**Conclusions:**

Fertility management in PCOS patients, especially in obese patients, shows significant gaps in adherence to recommended guidelines, highlighting the need for improved patient education, professional training and individualized treatment strategies. Improved health care is essential to address reproductive concerns and improve outcomes in this population.

**Supplementary Information:**

The online version contains supplementary material available at 10.1007/s00404-024-07916-1.

## What does this study add to the clinical work

**Table:** 

Modifiable factors such as weight management, improved patient education and counselling could improve treatment satisfaction and fertility outcomes. Needs of patients with obesity must be addressed more effectively.	

## Introduction

Polycystic ovary syndrome (PCOS) is the most common endocrine disorder in women of reproductive age, with a prevalence of 6–15%, and is the most common cause of anovulatory infertility [1, 2].

Obesity has been shown to exacerbate the reproductive, metabolic and psychological features of PCOS [3–5]. The prevalence of obesity in PCOS varies considerably between ethnic groups and is estimated to be 30–75% [4, 6]. Women with PCOS have higher rates of infertility, long-term weight gain and central obesity compared to women without PCOS [7, 8]. Because of these associations, recommendations for the management of subfertility associated with PCOS focus primarily on lifestyle changes rather than on pharmacotherapy, assisted reproductive technologies (ART) or surgical treatments [2, 9].

PCOS has a significant impact on the health-related quality of life (QoL) and psychological well-being of young women. Many PCOS patients are distressed by the concern that they might not be able to conceive. There is a general lack of knowledge about potential fertility problems and a great need for information among affected women [10].

The general dissatisfaction with the management of the psychological, metabolic and reproductive features of PCOS led to the development of the international evidence-based guideline for the assessment and management of PCOS, published in 2018 [2]. An update to the guideline in 2023 reinforces the recognition of the broader features of PCOS, including metabolic risk factors such as obesity [9].

Given the complexity of PCOS and its impact on multiple aspects of health and fertility, we suspected that PCOS patients, particularly those with obesity, may still not be receiving appropriate counselling to recommended standards despite current international guidelines. We therefore conducted a cross-sectional online survey of PCOS patients to assess their experience and satisfaction with medical care and to identify unmet clinical needs related to subfertility. Our aim was to gain insight into which aspects of healthcare professional training and patient information are adequate and which need to be improved.

## Material and methods

This cross-sectional cohort study in the form of an online survey was conducted in Germany, Austria and Switzerland from 2021-01-01 to 2021-03-14. The link to the online questionnaire was distributed on social media accounts of German speaking PCOS-related self-help forums and support groups, university newsletters and hospital websites. The study flyer and web link can be found in the supplementary section.

Women were included if they were 18 years of age or older, spoke German, had been diagnosed with PCOS by their physician, or had symptoms that met the revised Rotterdam ESHRE/ASRM consensus diagnostic criteria for PCOS [2, 11]. Women were excluded if they were postmenopausal or had been diagnosed with other causes of hyperandrogenism.

### Questionnaire

The development of the questionnaire (Supplementary file 1 and 2) was carried out in several steps, as previously published by our group [12, 13]. After a collaborative item collection and drafting of an initial questionnaire, we presented the template to 10 volunteers and adapted it according to individual feedback. After review by a statistician and a final review by all co-authors, the final questionnaire covered all areas of the ESHRE recommendations, including demographics, diagnostic criteria for PCOS (presence of two of the three diagnostic criteria: 1.) ovulatory dysfunction, 2.) clinical or biochemical hyperandrogenism and 3.) sonographically polycystic morphology in either ovary), aesthetic aspects, metabolic aspects, fertility and mental health. If a subject was affected by one or more of these aspects, more detailed questions were asked about subjective impairment and the patient's experience of treatment and counselling.

The current subgroup analysis looked at all women who completed the detailed fertility subquestionnaire and focused on childless women with a current desire to have children. If a participant had already tried at least one specific fertility treatment, she was asked about the number and type of treatments, treatment effectiveness, current fertility therapy, number of consultations, satisfaction with treatment consultations, and desire for further counselling. Finally, participants were asked to rate their overall satisfaction with fertility treatment and counselling on a scale of 0–100.

To reduce information bias, particularly recall bias due to self-assessment, the questions were asked in a neutral manner, participants were blinded to the study hypothesis, medical terms were explained, input validation was programmed and the option of ticking 'unknown' was provided. The questionnaire was programmed and executed in REDCap software to ensure secure data processing.

### Statistics

Descriptive statistics and statistical tests to compare groups, including chi-squared test, Student's t-test and Jonckheere-Terpstra test, were used to analyse the data as appropriate. A level of p < 0.05 was considered significant. As not all participants answered all questions, "n" is shown in the tables for each calculation. In a purely exploratory analysis, the effects of multiple testing were not considered. A backward stepwise linear regression model was used to identify relevant predictors of satisfaction while avoiding overfitting. Data were analysed using SPSS software version 29.0.

## Results

### Cohort characteristics

**Fig. 1:**
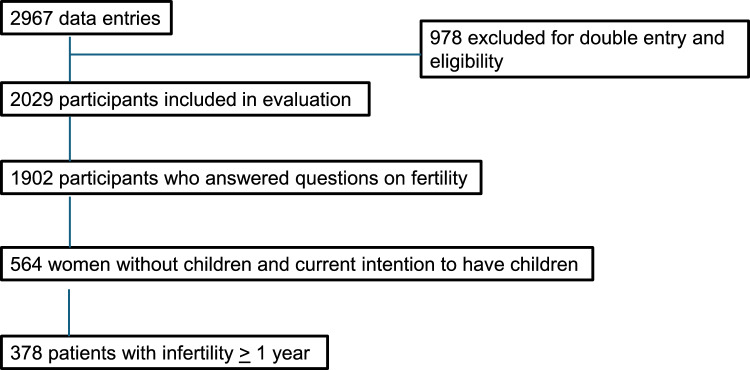
Flowchart on distribution of data entries

A total of 2967 records were recorded. After checking for duplicate entries and eligibility criteria, a total of 2029 participants were included in the final cohort (Fig. 1). 1720 women (84.8%) reported a diagnosis of PCOS by their health care provider (HCP), the remaining 309 women (15.2%) fulfilled the revised Rotterdam ESHRE/ASRM consensus diagnostic criteria for PCOS without previous physician's diagnosis. 1902 women (93.7%) completed the detailed fertility questionnaire. (Table 1). Most women lived in Germany (72.5%) and were educated (secondary school certificate or higher). The mean age was 28.9 ± 5.5 years. 41.0% of women were married. 29.5% women had already given birth to a child. 48.9% of women had a BMI ≥ 30 kg/m^2^. Menstrual irregularities were more frequent in obese patients (67.9% of patients with a BMI ≥ 30 kg/m^2^ vs. 62% of non-obese patients with a BMI < 30 kg/m^2^, p = 0.023). 20.3% of women reported cigarette smoking. More obese patients were smokers than non-obese women (26.0% vs. 14.8%, p < 0.001). Of the 1902 participants, 76.9% reported wanting to have children at some point in their lives. The frequencies were similar in obese (77.1%) and non-obese (76.6%) women. 73.9% women believed that their fertility was impaired. The belief that fertility was impaired was more common in obese women than in non-obese women (80.8% vs. 67.4%, p < 0.001).

**Table 1:** Characteristics of participants who answered questions on fertility (n = 1902)

Characteristics and responses		BMI < 30 kg/m^2^ (n = 972, 51.1%)	BMI ≥ 30 kg/m^2^ (n = 930, 48.9%)	Total^+^ (n = 1902)	P-value
Age	Mean (SD)	27.7 (SD 5.2)	30.2 (SD 5.4)	28.9 (SD 5.5)	** < 0.001**
Domicile	Switzerland	313 (32.2%)	65 (7.0%)	378 (19.9%)	
	Germany	586 (60.3%)	793 (85.3%)	1379 (72.5%)	
	Austria	59 (6.1%)	62 (6.7%)	121 (6.4%)	
	Other	14 (1.4%)	10 (1.1%)	24 (1.3%)	** < 0.001**
Civil state	Single or not in registered partnership	650 (68.4%)	399 (44.1%)	1049 (56.6%)	
	Married or in registered partnership	283 (29.8%)	478 (52.8%)	761 (41.0%)	
	Divorced or widowed	17 (1.8%)	28 (3.1%)	45 (2.4%)	** < 0.001**
Education	Secondary level 1, obligatory	128 (13.2%)	313 (33.7%)	441 (23.2%)	
	Secondary level 2, universal education	204 (21.0%)	120 (12.9%)	324 (17.0%)	
	Secondary level 2, vocational education	110 (11.3%)	179 (19.2%)	289 (15.2%)	
	Tertiary level, high vocational education	76 (7.8%)	103 (11.1%)	179 (9.4%)	
	Tertiary level, university	453 (46.6%)	215 (23.1%)	668 (35.1%)	
	Other	1 (.1%)	0 (.0%)	1 (.1%)	** < 0.001**
Smoking	No	828 (85.2%)	688 (74.0%)	1516 (79.7%)	
	Yes	144 (14.8%)	242 (26.0%)	386 (20.3%)	** < 0.001**
General intention to have children	No	103 (10.6%)	130 (14.0%)	233 (12.3%)	
	Yes	745 (76.6%)	717 (77.1%)	1462 (76.9%)	
	Unclear	124 (12.8%)	83 (8.9%)	207 (10.9%)	**0.004**
Having born a child	No	767 (78.9%)	574 (61.7%)	1341 (70.5%)	
	Yes	205 (21.1%)	356 (38.3%)	561 (29.5%)	** < 0.001**
Regular menstrual bleeding*	No	398 (62.0%)	491 (67.9%)	889 (65.1%)	
	Yes	244 (38.0%)	232 (32.1%)	476 (34.9%)	**0.023**
**Patient experience**					
Feeling of impaired fertility	No	317 (32.6%)	179 (19.2%)	496 (26.1%)	
	Yes	655 (67.4%)	751 (80.8%)	1406 (73.9%)	** < 0.001**
Desire for additional counselling	No	421 (43.4%)	314 (33.8%)	735 (38.7%)	
	Yes	550 (56.6%)	616 (66.2%)	1166 (61.3)	** < 0.001**
Overall satisfaction score	Mean (SD)	52.4 (SD 29.2)	46.5 (SD 31.9)	49.5 (SD 30.7)	** < 0.001**

*Cycle length within 28 + 7 days

### Current fertility concerns

We focused our analyses on a subgroup of 564 women who had not yet had children and were currently trying to have children. As shown in Table 2, the mean age of this cohort was 29.2 ± 4.1 years. 58.5% of these women were obese. 28.5% of women with a current desire to have children reported current smoking, 24.8% of normal and overweight women (BMI < 30 kg/m^2^) and 31.2% of obese women (BMI ≥ 30 kg/m^2^).

**Table 2:** Characteristics of participants without children and current intention to have children (n = 564)

Characteristics and responses		BMI < 30 kg/m^2^ (n = 234, 41.5%)	BMI ≥ 30 kg/m^2^ (n = 330, 58.5%)	Total (n = 564)	P-value
Age	Mean (SD)	28.9 (SD 3.8)	29.3 (SD 4.4)	29.2 (SD 4.1)	0.186
Smoking	No	176 (75.2%)	227 (68.8%)	403 (71.5%)	0.108
	Yes	58 (24.8%)	103 (31.2%)	161 (28.5%)	
Regular menstrual bleeding*	No	122 (67.0%)	213 (71.7%)	335 (69.9%)	0.305
	Yes	60 (33.0%)	84 (28.3%)	144 (30.1%)	
Infertility anytime (≥ 1 year)**	No	110 (47.0%)	76 (23.0%)	186 (33.0%)	** < 0.001**
	Yes	124 (53.0%)	254 (77.0%)	378 (67.0%)	
Actual pregnancy***	No	199 (86.1%)	314 (96.0%)	513 (91.9%)	** < 0.001**
	Yes	32 (13.9%)	13 (4.0%)	45 (8.1%)	
Patient experience					
Feeling of impaired fertility	No	23 (9.8%)	13 (3.9%)	36 (6.4%)	**0.008**
	Yes	211 (90.2%)	317 (96.1%)	528 (93.6%)	
Wish for possibility to ask more questions	No	128 (54.7%)	145 (43.9%)	273 (48.4%)	**0.013**
	Yes	106 (45.3%)	185 (56.1%)	291 (51.6%)	
Wish for more diagnostic tests	No	94 (40.2%)	101 (30.6%)	195 (34.6%)	**0.020**
	Yes	140 (59.8%)	229 (69.4%)	369 (65.4%)	
Desire for additional counselling	No	50 (21.4%)	55 (16.7%)	105 (18.6%)	0.188
	Yes	184 (78.6%)	275 (83.3%)	459 (81.4%)	
Overall satisfaction score	Mean (SD)	47.8 (SD 30.1)	40.9 (SD 32.4)	43.7 (SD 31.6)	**0.009**

^*^Cycle length within 28 + 7 days

^**^ Answer: “yes” to question: Have you ever tried to get pregnant for (≥ 1 year)

^***^ Answer: “yes” to question: Are you pregnant ?

**Fig. 2:**
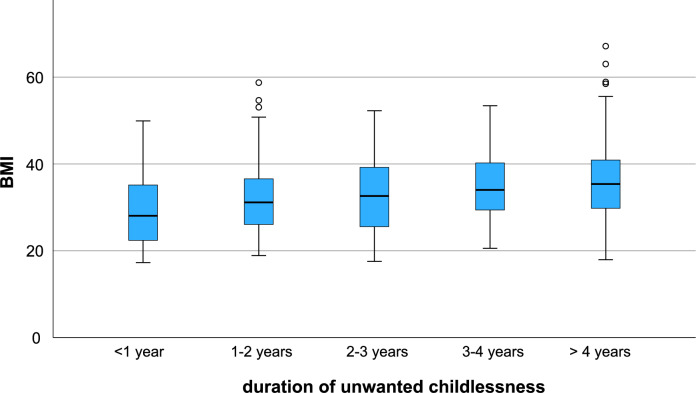
Association of duration of infertility with BMI. Box plots showing the distribution of BMI values among infertility patients grouped according to their duration of unwanted childlessness, demonstrating a positive association (Spearman correlation coefficient 0.274, p < 0.001)

Overall, 93.6% of women in this group felt that their fertility was affected. The subjective perception of reduced fertility was significantly more common in the obese compared to non-obese patients (96.1% vs. 90.2%, p = 0.008). There were also significantly fewer current pregnancies in obese PCOS patients compared to non-obese participants (4% vs. 13.9%, p < 0.001). In addition, obese patients were significantly more likely to report infertility or failure to conceive after one year or more. (77.0% vs. 53.0%, p < 0.001). Finally, a significant correlation was also found between BMI as a continuous parameter and the duration of unwanted childlessness, starting with a median BMI of 28.0 kg/m^2^ for less than 1 year of infertility and increasing steadily to a median BMI of 35.4 kg/m^2^ for more than 4 years of infertility (p < 0.001, Jonckheere-Terpstra test) (Fig. 2).

### Infertility treatment

Of the 564 childless women who were currently trying to become pregnant, 378 (67.0%) answered 'yes' when asked if they had been trying unsuccessfully to become pregnant for more than a year, thus fulfilling the WHO criteria for infertility. There were no significant differences in this subgroup regarding smoking or menstrual irregularities (Table 3). However, the number of obese women was significantly higher in this group (67.2% compared to 40.9% in the group of women without diagnosed infertility, p < 0.001). The 378 patients with infertility according to WHO criteria were then asked about fertility treatment. 63.0% received this type of treatment (Table 4). When asked how fertility treatment was being carried out, only 34.1% of infertile patients reported that lifestyle changes were part of the treatment. Lifestyle adjustments included, for example, specific measures to increase physical activity (28.3% of infertile women), weight loss (28.6%) or dietary changes (33.1%). The use of the insulin sensitizer metformin was reported by 34.9% of infertile patients. According to patient reports, the selective estrogen receptor modulator clomiphene citrate, recommended for years as first-line treatment for anovulatory infertility, was the most used fertility drug (35.4%). The aromatase inhibitor letrozole, currently considered first-line treatment, was used in only 14.6% of cases. The use of gonadotropins, which are recommended as second-line treatment in the international ESHRE PCOS guideline, was reported by 24.6% of patients. 11.9% were treated with in vitro fertilization (IVF) or intracytoplasmic sperm injection (ICSI). As shown in Table 5, obese patients were significantly less likely to receive fertility treatment compared to non-obese patients (56.7% vs. 75.8%, p < 0.001). There was no significant difference between obese and non-obese patients with regard to lifestyle modification as a fertility-optimizing measure.

**Table 3:** Characteristics of participants according to WHO criteria for infertility

		Have you ever tried to get pregnant for over a year without success?			
		No n = 186 (33.0%)	Yes n = 378 (67.0%)	Total (n = 564)	P-value
Age	Mean (SD)	28.0 (SD 4.0)	29.7 (SD 4.1)	29.2 (SD 4.1)	** < 0.001**
BMI	< 30 kg/m^2^	110 (59.1%)	124 (32.8%)	234 (41.5%)	** < 0.001**
	≥ 30 kg/m^2^	76 (40.9%)	254 (67.2%)	330 (58.5%)	
Regular menstrual bleeding*	No	107 (74.3%)	228 (68.1%)	335 (69.9%)	0.193
	Yes	37 (25.7%)	107 (31.9.%)	144 (30.1%)	
Smoking	No	140 (75.3%)	263 (69.6%)	403 (71.5%)	0.166
	Yes	46 (24.7%)	115 (30.4%)	161 (28.5%)	
Patient experience					
Desire for additional counselling	No	54 (29.0%)	51 (13.5%)	105 (18.6%)	** < 0.001**
	Yes	132 (71.0%)	327 (86.5%)	459 (81.4%)	
Overall satisfaction score	Mean (SD)	46.2 (SD 30.0)	42.4 (SD 32.3)	43.7 (SD 31.6)	**0.172**

*Cycle length within 28 + 7 days

**Table 4:** Fertility treatment among participants with WHO criteria for infertility (n = 378)

	Number (%) of n = 378 participants with infertility
**Infertility treatment**	**238 (63.0%)**
**Lifestyle adaptation**	**129 (34.1%)**
Behavioural interventions	68 (18.0%)
Attitude	41 (10.8%)
Dietary interventions	125 (33.1%)
Physical activity	107 (28.3%)
Weight assessment and reduction	108 (28.6%)
Blood glucose regulation	49 (13.0%)
Blood pressure regulation	10 (2.6%)
Abstinence from smoking, alcohol, drugs	72 (19.0%)
Sleep regulation	38 (10.1%)
Changes regarding sexual life	54 (14.3%)
others	0 (0.0%)
**Medication**	**206 (54.5%)**
Letrozole	55 (14.6%)
Clomiphene	134 (35.4%)
Metformin	132 (34.9%)
Gonadotropins	93 (24.6%)
Others	22 (5.8%)
Unknown	2 (0.5%)
**Surgery**	**47 (12.4%)**
**Artificial reproductive technologies (ART)**	**45 (11.9%)**
**Others**	**13 (3.4%)**

**Table 5:** Treatment among participants with WHO criteria for infertility (n = 378) in association with BMI

Characteristics and responses		BMI < 30 kg/m^2^ (n = 124, 32.8%)	BMI ≥ 30 kg/m^2^ (n = 254, 67.2%)	Total^+^ (n = 378)	P-value
Infertility treatment	No	30 (24.2%)	110 (43.3%)	140 (37.0%)	** < 0.001**
	Yes	94 (75.8%)	144 (56.7%)	238 (63.0%)	
Lifestyle adaptation	No	78 (62.9%)	171 (67.3%)	249 (65.9%)	0.420
	Yes	46 (37.1%)	83 (32.7%)	129 (34.1%)	

### Counselling

Of the 1902 patients who completed the fertility questionnaire, 61.3% expressed a desire for further counselling. This was significantly more common in obese compared to non-obese patients (66.2% vs. 56.6%, p < 0.001) (Table 1). Of the 564 patients who were childless and currently wished to have children, 81.4% wished for additional counselling (Table 2) and 51.6% wished they had had more opportunities to ask questions. The latter wish was significantly more common in obese compared to non-obese patients (56.1% vs. 45.3%, p = 0.013) (Table 2). In addition, 65.4% of childless patients with a current desire to have children would have liked more fertility testing. This finding was also significantly more common in obese compared to non-obese patients (69.4% vs. 59.8%, p = 0.020) (Table 2). The desire for further counselling was also significantly more common among the 378 patients who had been infertile for more than one year than among women who were not infertile (86.5% vs. 71.0%, p < 0.001) (Table 3).

### Overall satisfaction with fertility treatment

When patients were asked to rate their overall satisfaction with fertility treatment and counselling on a scale of 0–100, the mean score of all 1902 patients who completed the fertility questionnaire was 49.5 (SD 30.7). Satisfaction was significantly lower in patients with obesity versus patients without obesity (46.5 vs. 52.4, p < 0.001) (Table 1). In the 564 patients who were childless and currently wished to have children, the mean satisfaction score was 43.7 (SD 31.6). Again, satisfaction was significantly lower in obese patients (40.9 vs. 47.8, p = 0.009) (Table 2). Again, we used a stepwise backward multivariate regression model to predict satisfaction in the 564 cases without a child and current desire to have children, based on 70 parameters, including age, BMI, duration of infertility, menstrual regularity, previous pregnancies, employment status, cardiovascular risk factors or diseases, previous and current infertility treatments, and desire for additional counselling (Supplementary Table 1). Thirteen parameters remained as independent factors in the model and are shown in Table 6. A significant negative influence on satisfaction was found for higher BMI, unemployment, smoking, current surgical treatment and desire for additional counselling and treatment options. A positive influence on satisfaction was found for some specific infertility treatments (e.g. lifestyle, glucose level adjustment, clomiphene treatment and oocyte stimulation) (Table 6).

**Table 6:** Multivariate regression model for prediction of satisfaction with treatment (among participants without children and current intention to have children)

Parameter	Coefficient B	(95% CI)	p-value
BMI (continuous)	− 0.417	(− 0.685 … − 0.149)	0.002
Regular menstrual bleeding	− 5.096	(− 10.372 … 0.180)	0.058
Unemployment	− 12.336	(− 22.730 … − 1.943)	0.020
Smoking	− 5.936	(− 11.038 … − 0.834)	0.023
Infertility treatment: Blood glucose regulation	9.069	(0.411 … 17.727)	0.040
Infertility treatment: Medication Clomiphene	7.569	(1.607 … 13.531)	0.013
Infertility treatment: Medication unknown	56.280	(19.945 … 92.614)	0.002
Current treatment: any medical ovarian stimulation	8.278	(1.792 … 14.764)	0.012
Current treatment: Surgery	− 25.869	(− 49.479 … − 2.260)	0.032
Desire for additional counselling	− 23.827	(− 34.758 … − 12.896)	0.000
Desire for more reassurance	− 7.273	(− 15.094 … 0.549)	0.068
Desire for possibility to ask questions	− 7.041	(− 12.687 … − 1.395)	0.015
Desire for more therapeutic possibilities	− 7.915	(− 15.221 … − 0.609)	0.034

## Discussion

Our study confirms that fertility is a pressing concern for patients with polycystic ovary syndrome (PCOS). Overall, 76.9% of women with PCOS expressed a desire to have children and 73.9% felt that their fertility was affected by PCOS. In fact, 67.0% of women suffered from infertility according to World Health Organization (WHO) criteria. The need for comprehensive counselling is clear: 61,3% of all participants and 86.5% of those with infertility wanted more counselling.

A key finding of our study is the high prevalence of obesity, with 48.9% of participants having a body mass index (BMI) ≥ 30 kg/m^2^. The prevalence of obesity in our cohort is consistent with a systematic review and meta-analysis of 21 studies, which found a pooled prevalence of overweight or obesity in women with PCOS of 61% [14]. Obesity is relevant because it correlates with fewer pregnancies and longer periods of infertility. In addition, obese patients were less likely to report fertility counselling and treatment than non-obese women, which is consistent with lower satisfaction with fertility treatment in this group. This suggests a significant unmet need for specialist fertility treatment, particularly for obese PCOS patients.

Our review of treatment practices found that infertility treatment for PCOS patients does not strictly follow guideline recommendations. For example, letrozole is the recommended first-line treatment for anovulation, but clomiphene citrate (35.4%) was prescribed more often than letrozole (14.6%). In addition, only 34.1% of infertility patients reported lifestyle modification as part of PCOS and infertility treatment, despite this being recommended as a first-line treatment for obese PCOS patients. This discrepancy suggests that the implementation of lifestyle modification as a standard treatment is not yet well established.

The strengths of our study are the large number of participants and the anonymous, independently administered survey, which minimizes potential bias.

However, the study relies extensively on self-reported data, making it susceptible to recall bias. This may compromise the accuracy of patient-reported fertility status, the duration of infertility, and satisfaction with treatment. Respondents could either overestimate or underestimate the length of time they have faced infertility or the influence of obesity on their fertility. In addition, the study used a non-standardized and non-validated questionnaire, which raises concerns about the reliability and consistency of the data. A validated tool would help minimize potential biases related to question phrasing and interpretation, which is particularly important in studies relying on subjective patient reports. Finally, not all questions were answered by all participants, which limits statistical power.

Comparisons with the existing literature show that our results are consistent with previous studies. For example, an Australian study found a 15-fold increased risk of infertility in women with PCOS, independent of BMI [15]. Other studies have confirmed that fertility problems and concerns about fertility are common in young women with PCOS and have a negative impact on their QoL [10, 16, 17]. The prevalence of obesity in our cohort is consistent with a systematic review and meta-analysis of 21 studies, which found a pooled prevalence of overweight or obesity in women with PCOS of 61% [14].

A revised version of the guideline was published in 2023; However, the content concerning fertility and obesity, which is relevant to our study, remains unchanged. In the recently updated PCOS guideline, Teede et al. highlight the need for improved healthcare, professional education, evidence-based patient information, better care models and shared decision-making to improve patient experience [9].

Our findings highlight the need for improved implementation of these guideline recommendations in clinical practice.

The low number of women (14.6%) using letrozole for ovulation induction suggests limited adherence to the 2018 guideline recommendation. It must be noted, that at the time of our survey, recommendations were less robust than they are today. Physicians had only just begun prescribing letrozole off-label.

However, only 34.1% of women reported making lifestyle changes, even though such changes are considered highly effective.

Our multivariate regression analysis identified factors which independently influenced satisfaction with fertility treatment. Higher BMI, unemployment, smoking and desire for additional counselling were negatively associated with satisfaction, whereas lifestyle changes and clomiphene treatment had a positive influence. These findings could suggest that modifiable factors such as weight management and smoking cessation, as well as improved patient education and counselling, might improve treatment satisfaction. However, causality remains to be clarified and the current study does not address potential interventions or examine the challenges healthcare providers face in treating this population.

In conclusion, our study highlights significant gaps in the management of subfertility in women with PCOS, particularly in obese patients. The underuse of lifestyle interventions and dissatisfaction with current counselling and treatment highlight the need for better adherence to clinical guidelines and the development of more effective, individualized treatment strategies. Future research should focus on longitudinal studies to better understand the causal relationships between obesity, lifestyle interventions and fertility outcomes in PCOS, and the development of interventions that integrate metabolic and reproductive management to improve quality of life and treatment satisfaction in these patients.

## Supplementary Information

Below is the link to the electronic supplementary material.

Supplementary file1 (PDF 905 KB)

Supplementary file2 (PDF 347 KB)

Supplementary file3 (PDF 665 KB)

Supplementary file4 (PDF 111 KB)
