# Supplementary material for: Unmet clinical needs in women with polycystic ovary syndrome regarding fertility and obesity: a cross-sectional study from the patient’s perspective
Source: Arch Gynecol Obstet. 2025 Jan 25;311(3):851–9. doi: 10.1007/s00404-024-07916-1 (PMC11920305; doi:10.1007/s00404-024-07916-1)
Supplement: Supplementary file 2 — Supplementary file2 (PDF 347 KB) [file 404_2024_7916_MOESM2_ESM.pdf]

Einverständnis

"Versorgungssituation und -bedarf bei Frauen mit Polyzystischem Ovarialsyndrom"

Herzlich Willkommen bei dieser Umfrage!

Bitte lesen Sie die untenstehenden Informationen sorgfältig durch und geben Sie danach Ihr Einverständnis, um mit der Umfrage zu beginnen.

Was ist ein PCOS?

PCOS (Polyzystisches Ovarialsyndrom) ist die häufigste hormonelle Erkrankung bei Frauen im geschlechtsreifen Alter. Zu den typischen Symptomen zählen seltene bis gar keine Menstruationsblutungen und «Vermännlichungszeichen» wie Haarausfall, Zunahme der Körperbehaarung und Akne.

Warum führen wir diese Studie durch?

Das Ziel dieser Studie ist zu untersuchen, wie gut die neuen Leitlinien zur Betreuung von Frauen mit PCOS (Polyzystischem Ovarialsyndrom) von Frauenärztinnen/-ärzten umgesetzt werden. Ausserdem interessiert es uns, in welchen Bereichen sich Frauen mit PCOS zusätzliche Betreuung wünschen würden. Dazu ist Ihre ehrliche Meinung gefragt.

Teilnahmekriterien:

An der Studie teilnehmen können alle deutschsprachigen Frauen mit PCOS, die volljährig sind und noch nicht die Menopause erreicht haben. Wenn Sie noch keine Diagnose PCOS erhalten haben, aber anhand der Symptome vermuten, dass Sie betroffen sein könnten, dann können Sie gerne auch teilnehmen! Falls Sie die Kriterien nicht erfüllen sollten, wird Ihnen dies im ersten Teil der Umfrage signalisiert und die Umfrage wird beendet.

Was haben Sie davon?

Sie leisten einen wichtigen Beitrag zur Verbesserung der Versorgungssituation von Frauen mit PCOS. Falls Sie Interesse an den Resultaten der Studie haben, wird Ihnen am Ende der Umfrage angezeigt, wie das funktioniert.

Ethik und Anonymität:

Die Studie wurde von den zuständigen Ethikkommissionen als unbedenklich eingestuft. Es werden Fragen zu Ihrer Gesundheit gestellt, aber keine persönliche, identifizierbare Daten erhoben. Die Daten werden also anonym erfasst. Somit können Ihre Antworten nicht auf Sie zurückgeführt werden. Stattdessen werden Sie auf der ersten Seite der Umfrage angeleitet, einen persönlichen Code zu kreieren. Damit wird sichergestellt, dass jede Person nur einmal an der Umfrage teilnimmt.

Wichtige Infos:

Die Umfrage dauert ungefähr 15-20 Minuten. Sie können die Teilnahme jederzeit und ohne Angabe von Gründen abbrechen. Wir möchten Sie darauf hinweisen, dass wir nur vollständig ausgefüllte Umfragen für die Studie verwenden können. Bitte lesen Sie die Fragen genau und beantworten Sie sie so korrekt wie möglich. Unterhalb des Antwortfeldes finden Sie teilweise hilfreiche Zusatzinformationen oder Anleitungen.

Falls Sie einverstanden sind, klicken Sie auf "Ja" und die Umfrage kann beginnen.

Wir danken Ihnen herzlich für Ihre Teilnahme!

1) Sind Sie mit der Umfrage einverstanden?
- ◯ Ja
- ◯ Nein

Klicken Sie jeweils auf "Submit", um zur nächsten Seite zu gelangen.

Charakteristika

Bitte beantworten Sie die untenstehenden Fragen.

Herzlichen Dank!

Teilnehmer-ID

Bilden Sie bitte ihre persönliche ID in GROSSBUCHSTABEN aus folgenden Angaben:
- erster und letzter Buchstabe des Vornamens Ihrer Mutter
- erster und letzter Buchstabe des Vornamens Ihres Vaters
- erster und letzter Buchstabe Ihres Vornamens
- letzte zwei Ziffern Ihres Geburtsjahres

Ein Beispiel finden Sie unter dem Antwortfeld. Können Sie eine der Angaben nicht machen (z.B. wissen Sie den Namen eines Elternteils nicht), dann schreiben Sie an dieser Stelle "XX".

____
(Bsp: AETNMA76 (Mutter Anne, Vater Thorsten, Sie Michaela, geboren 1976))

Später fortsetzen: Sie können die Umfrage jederzeit unterbrechen und zu einem späteren Zeitpunkt fortsetzen. Klicken Sie dazu auf "Safe & Return Later". Sie erhalten dann einen Code, womit Sie später an derselben Stelle fortfahren können. Die bereits beantworteten Fragen bleiben dabei gespeichert.

Charakteristika

Geschlecht

◯ weiblich
◯ männlich
◯ andere

Geschlecht - andere

____
(Bitte genauer ausführen.)

Leider erfüllen Sie die Kriterien zur Teilnahme an dieser Studie nicht. Wir danken Ihnen trotzdem für Ihre Bereitschaft und wünschen Ihnen alles Gute. Bitte klicken Sie auf "submit". Danach können Sie die Seite schliessen.

Alter

____

Leider erfüllen Sie die Kriterien zur Teilnahme an dieser Studie nicht. Wir danken Ihnen trotzdem für Ihre Bereitschaft und wünschen Ihnen alles Gute. Bitte klicken Sie auf "submit". Danach können Sie die Seite schliessen.

Grösse [cm]

____
(Bsp: 165)

Gewicht [kg]

____
(Bsp: 80)

BMI

____
(Wird automatisch berechnet.)

Wohnort

◯ Schweiz
◯ Deutschland
◯ Österreich
◯ Andere
(In welchem Land wohnen Sie?)

Wohnort - Andere

____
(Bitte genauer ausführen.)

Anzahl Kinder

◯ keine
◯ 1
◯ 2
◯ 3
◯ 4
◯ 5
◯ 6
◯ 7
◯ 8
◯ 9
◯ 10
◯ mehr als 10

Zivilstand	◯ ledig<br>◯ verheiratet<br>◯ in eingetragener Partnerschaft<br>◯ verwitwet<br>◯ geschieden<br>◯ andere/keine Angabe
Bildungsstand:<br>Bitte wählen Sie Ihren höchsten Abschluss aus.	◯ kein Abschluss<br>◯ Sekundarstufe I (=obligatorische Schule: Sekundarschule, Realschule, Bezirksschule)<br>◯ Sekundarstufe II Allgemeinbildung (gymnasiale Maturität, Fachmittelschule)<br>◯ Sekundarstufe II Berufsbildung (EBA-Anlehre, EFZ-Lehre, Berufsmaturität)<br>◯ Tertiärstufe höhere Berufsbildung (höhere Fachschule HF z.B. HWV, HFG, HFS, Ingenieursschule HTL)<br>◯ Tertiärstufe Hochschule (Universität, Fachhochschule, pädagogische Hochschule)<br>◯ Andere
Bildungsstand:<br>Bitte wählen Sie Ihren höchsten Abschluss aus.	◯ kein Abschluss<br>◯ Sekundarstufe I (=obligatorische Schule: Gymnasium, Gesamtschule, Realschule, Hauptschule)<br>◯ Sekundarstufe II Allgemeinbildung (gymnasiale Oberstufe mit Abitur)<br>◯ Sekundarstufe II Berufsbildung (berufliche Schule z.B. BFS, FGY; duales System)<br>◯ Tertiärstufe höhere Berufsbildung (Fachschule, Fachakademie)<br>◯ Tertiärstufe Hochschule (Universität, Fachhochschule, Berufsakademie, duale Hochschule)<br>◯ Andere
Bildungsstand:<br>Bitte wählen Sie Ihren höchsten Abschluss aus.	◯ kein Abschluss<br>◯ Sekundarstufe I (=obligatorische Schule; Neue Mittelschule (NMS), allgemein bildende höhere Schule (AHS) Unterstufe)<br>◯ Sekundarstufe II Allgemeinbildung (allgemein bildende höhere Schule (AHS) Oberstufe mit Maturität)<br>◯ Sekundarstufe II Berufsbildung (integrative Berufsausbildung, Berufsschule und Lehre, berufsbildende mittlere/höhere Schule)<br>◯ Tertiärstufe höhere Berufsbildung (Aufbaulehrgang, Werkmeister-, Bauhandwerker- und Meisterschule, Kolleg, Schule für Berufstätige)<br>◯ Tertiärstufe Hochschule (Universität, Fachhochschule, pädagogische Hochschule)<br>◯ Andere

Bildungsstand:<br>Bitte wählen Sie Ihren höchsten Abschluss aus.	◯ kein Abschluss<br>◯ Sekundarstufe I (=obligatorische Schule: Sekundarschule, Realschule, Bezirksschule)<br>◯ Sekundarstufe II Allgemeinbildung (gymnasiale Maturität, Fachmittelschule)<br>◯ Sekundarstufe II Berufsbildung (EBA-Anlehre, EFZ-Lehre, mit/ohne Berufsmaturität)<br>◯ Tertiärstufe höhere Berufsbildung (höhere Fachschule HF z.B. HWV, HFG, HFS, Ingenieursschule HTL)<br>◯ Tertiärstufe Hochschule (Universität, Fachhochschule, pädagogische Hochschule)<br>◯ Andere
Bildungsstand - Andere	______________________________<br>(Bitte genauer ausführen.)
Wie viele Jahre haben Sie die Schule besucht?	__________________________
Erwerbsstatus:<br>Bitte kreuzen Sie ALLE Zutreffenden an.	☐ Vollzeit (100%)<br>☐ Teilzeit<br>☐ im Stundenlohn<br>☐ Studentin / in Ausbildung<br>☐ unbezahltes Praktikum<br>☐ Selbständig<br>☐ nicht-angestellt / arbeitslos<br>☐ Andere
In welchem Pensum sind Sie Teilzeit angestellt? [%]	◯ 5%<br>◯ 10%<br>◯ 15%<br>◯ 20%<br>◯ 25%<br>◯ 30%<br>◯ 35%<br>◯ 40%<br>◯ 45%<br>◯ 50%<br>◯ 55%<br>◯ 60%<br>◯ 65%<br>◯ 70%<br>◯ 75%<br>◯ 80%<br>◯ 85%<br>◯ 90%<br>◯ 95%
Erwerbsstatus - Andere	______________________________<br>(Bitte genauer ausführen.)

Abstammung / Hautfarbe (Mehrfachauswahl möglich)	☐ Weiss (Kaukasier/Hellhäutige)<br>☐ Mediterran (Frankreich; Portugal; Spanien; Italien; Griechenland; Malta; Zypern)<br>☐ Hispanisch (Amerikaner spanischer oder lateinamerikanischer Herkunft)<br>☐ Naher Osten (Arabische Länder, Iran, Türkei)<br>☐ Süd-Asien (Afghanistan, Bangladesch, Bhutan, Indien, Malediven, Nepal, Pakistan, Sri Lanka)<br>☐ Ost-Asien (China, Hong Kong, Macau, Taiwan, Japan, Nordkorea, Südkorea, Mongolei)<br>☐ Süd-Ost-Asien (Brunei, Kambodscha, Indonesien, Laos, Malaysia, Myanmar, Philippinen, Singapur, Thailand, Osttimor, Vietnam)<br>☐ Aborigines (=australische Ureinwohner)<br>☐ Schwarz<br>☐ Andere<br>(Bitte wählen Sie die ethnische Zugehörigkeit, die Ihrer Meinung nach am besten passt. Es ist auch möglich mehrere anzukreuzen, wenn beispielsweise Ihre Eltern aus verschiedenen Regionen stammen.)
Abstammung und Hautfarbe - Andere	__________________________________<br>(Bitte genauer ausführen.)
Aus welcher Region stammen Sie?	☐ Europa<br>☐ Australien<br>☐ Nordamerika<br>☐ Andere<br>(Bitte ordnen Sie Ihre Abstammung genauer ein. Es ist auch möglich mehrere anzukreuzen, wenn beispielsweise Ihre Eltern aus verschiedenen Regionen stammen.)
Region - andere	__________________________________<br>(Bitte genauer ausführen.)

Diagnosekriterien - Teil 1

Bitte beantworten Sie die untenstehenden Fragen.

Herzlichen Dank!

Menstruationszyklus

Wissen Sie, wie alt Sie bei Ihrer ersten Menstruation waren?	◯ Alter (ungefähr) bekannt<br>◯ Alter unbekannt<br>◯ noch nie menstruiert

Vor wie vielen Jahren hatten Sie Ihre erste Menstruation?<br><br>Rechnen Sie dazu Ihr jetziges Alter minus das Alter bei Ihrer ersten Menstruation.	◯ 1<br>◯ 2<br>◯ 3<br>◯ 4<br>◯ 5<br>◯ 6<br>◯ 7<br>◯ 8<br>◯ 9<br>◯ 10<br>◯ 11<br>◯ 12<br>◯ 13<br>◯ 14<br>◯ 15<br>◯ 16<br>◯ 17<br>◯ 18<br>◯ 19<br>◯ 20<br>◯ 21<br>◯ 22<br>◯ 23<br>◯ 24<br>◯ 25<br>◯ 26<br>◯ 27<br>◯ 28<br>◯ 29<br>◯ 30<br>◯ 31<br>◯ 32<br>◯ 33<br>◯ 34<br>◯ 35<br>◯ 36<br>◯ 37<br>◯ 38<br>◯ 39<br>◯ 40<br>◯ 41<br>◯ 42<br>◯ 43<br>◯ 44<br>◯ 45<br>◯ 46<br>◯ 47<br>◯ 48<br>◯ 49<br>◯ 50<br>(Bitte die Zahl in Jahren angeben.)
Sind Sie schon in der Menopause?	◯ Ja<br>◯ Nein<br>(Die Menopause ist dann erreicht, wenn bei über 40-jährigen Frauen seit mindestens 12 Monaten keine Menstruation mehr erfolgte.)
Seit wie vielen Jahren haben Sie keine Periode mehr?	______________________________

Leider erfüllen Sie die Kriterien zur Teilnahme an dieser Studie nicht. Wir danken Ihnen trotzdem für Ihre Bereitschaft und wünschen Ihnen alles Gute. Bitte klicken Sie auf "submit". Danach können Sie die Seite schliessen.

Mit den folgenden Fragen soll die Regelmässigkeit Ihrer Periode ermittelt werden. Dazu werden Fragen zur Zyklusdauer und zur Periodendauer gestellt. Wenn Sie nicht genau wissen, wie viele Tage Ihr Zyklus oder Ihre Periode dauert, geben Sie eine ungefähre Zahl an.

- Zyklusdauer= Anzahl Tage zwischen 1.Tag der Menstruation und 1.Tag der nächsten Menstruation

- Periodendauer= Anzahl Tage an denen Sie bluten

Aktuelle Periode

Die folgenden Fragen beziehen sich auf Ihre aktuelle Periode. Denken Sie bei der Beantwortung dieser Fragen nur an die letzten 12 Monate.

Falls Sie zur Zeit hormonell verhüten (Pille, Hormonspirale, Stäbchen, Vaginalring, etc.) oder aktuell schwanger sind werden Ihnen keine weiteren Fragen zu der aktuellen Periode gestellt.

Verhüten Sie momentan mit einer hormonellen Methode?	◯ Ja<br>◯ Nein<br>(z.B. Pille, Hormonspirale, Stäbchen, Vaginalring, etc.)
Sind Sie im Moment schwanger oder stillen?	◯ Ja<br>◯ Nein
Dauert Ihr Zyklus immer ungefähr gleich lang (+/- 7 Tage)?	◯ Ja<br>◯ Nein<br>(Bsp. im April dauert ein Zyklus 27 Tage, im Mai 33 Tage, Unterschied = 6Tage -> JA)
Wie lange dauert Ihr Zyklus durchschnittlich? [Tage]	________________<br>(Anzahl Tage vom 1.Tag der Menstruation bis zum 1.Tag der nächsten Menstruation. Der Durchschnittswert beträgt 28 Tage.)
Wie lange dauert Ihre Periode durchschnittlich? [Tage]	________________<br>(Anzahl Tage, an denen Sie bluten.)
Inwiefern dauert Ihr Zyklus nicht immer ungefähr gleich lang?	◯ keine Periode<br>◯ Schwankung der Zyklusdauer >8 Tage<br>◯ andere
Seit wie vielen Monaten hatten Sie keine Periode mehr? [Monate]	________________<br>(Bitte geben Sie die Antwort in Monaten. Wenn es mehrere Jahre sind, rechnen Sie die Anzahl Jahre bitte mal 12.)
Was war in den letzten 12 Monaten die kürzeste Dauer Ihres Zyklus? [Tage]	________________<br>(Anzahl Tage vom 1.Tag der Menstruation bis zum 1.Tag der nächsten Menstruation)
Was war in den letzten 12 Monaten die längste Dauer Ihres Zyklus? [Tage]	________________<br>(Anzahl Tage vom 1.Tag der Menstruation bis zum 1.Tag der nächsten Menstruation)

Andere Unregelmässigkeit

____
(Bitte genauer beschreiben.)

Wie lange dauert Ihre Periode durchschnittlich? [Tage]

____
(Anzahl Tage, an denen Sie bluten.)

Wie viele Menstruationszyklen haben Sie durchschnittlich pro Jahr?

____

Frühere Periode

Die folgenden Fragen beziehen sich auf Ihre Periode in der Vergangenheit. Denken Sie bei der Beantwortung dieser Fragen an die Zeit vor mehr als 12 Monaten.

Uns interessiert hier Ihr natürlicher Zyklus. Ignorieren Sie deshalb das erste Jahr nach Ihrer allerersten Menstruation. Im ersten Jahr nach der ersten Menstruation sind Schwankungen völlig normal. Geben Sie solche Schwankungen hier nicht an. Ebenfalls nicht beachten sollen Sie die Zeit von Schwangerschaft, Stillen und hormoneller Verhütung.

Falls Sie hormonell verhüten oder verhütet haben (Pille, Hormonspirale, Stäbchen, Vaginalring, etc.), beantworten Sie die Fragen für die Zeit vor der hormonellen Verhütung.

Hatten Sie schon einmal einen Zyklus, der mehr als 90 Tage dauerte?

◯ Ja
◯ Nein

Dauerte Ihr Zyklus früher immer ungefähr gleich lang (+/- 7 Tage)?

◯ Ja
◯ Nein
(Bsp. im April dauert ein Zyklus 27 Tage, im Mai 33 Tage, Unterschied = 6Tage -> JA)

Für ungefähr wie viele Jahre war Ihre Periode regelmässig? [Jahre]

____
(Eine ungefähre Schätzung reicht hier.)

Seit ungefähr wie vielen Jahren haben Sie eine unregelmässige Periode? [Jahre]

____
(Eine ungefähre Schätzung reicht hier.)

Wie lange dauerte Ihr Zyklus damals durchschnittlich (ungefähr)? [Tage]

____
(Anzahl Tage vom 1.Tag der Menstruation bis zum 1.Tag der nächsten Menstruation. Der Durchschnittswert beträgt 28 Tage.)

Wie lange dauerte Ihre Periode damals durchschnittlich? [Tage]

____
(Anzahl Tage, an denen Sie geblutet haben.)

Für ungefähr wie viele Jahre war Ihre Periode unregelmässig? [Jahre]

____

Seit ungefähr wie vielen Jahren haben Sie eine regelmässige Periode? [Jahre]

____

Inwiefern dauerte Ihr Zyklus nicht immer ungefähr gleich lang?	◯ keine Periode<br>◯ Schwankung der Zyklusdauer >8 Tage<br>◯ andere
Für ungefähr wie vielen Monate hatten Sie keine Periode mehr? [Monate]	__________________________________<br>(Bitte geben Sie die Antwort in Monaten. Wenn es mehrere Jahre sind, rechnen Sie die Anzahl Jahre bitte mal 12.)
Was war die kürzeste Dauer eines Zyklus, an die Sie sich erinnern können? [Tage]	__________________________________
Was war die längste Dauer eines Zyklus, an die Sie sich erinnern können? [Tage]	__________________________________
Andere Unregelmässigkeit	____<br>(Bitte genauer beschreiben.)
Wie lange dauerte Ihre Periode damals durchschnittlich? [Tage]	__________________________________<br>(Anzahl Tage, an denen Sie geblutet haben.)
Wie viele Menstruationszyklen hatten Sie damals durchschnittlich pro Jahr?	__________________________________
Ist der Beginn Ihrer Brustentwicklung schon länger als 3 Jahre her?	◯ Ja<br>◯ Nein

Ihre Periode gilt als unregelmässig.

Diagnosekriterien - Teil 2

Bitte beantworten Sie die untenstehenden Fragen.

Herzlichen Dank!

Klinischer Hyperandrogenismus

Leiden oder litten Sie je an Akne?	◯ Ja<br>◯ Nein<br>◯ Unbekannt
Leiden oder litten Sie je an übermässigem Haarausfall? Betrachten Sie dazu das folgende Bild.	◯ Ja<br>◯ Nein<br>◯ Unbekannt

Damit Sie sich etwas unter "übermässigem Haarausfall" vorstellen können, sehen Sie dieses Bild mit verschiedenen Schweregraden des Haarausfalls. Wenn eins davon auf Sie zutrifft, beantworten Sie die Frage oben mit "JA".

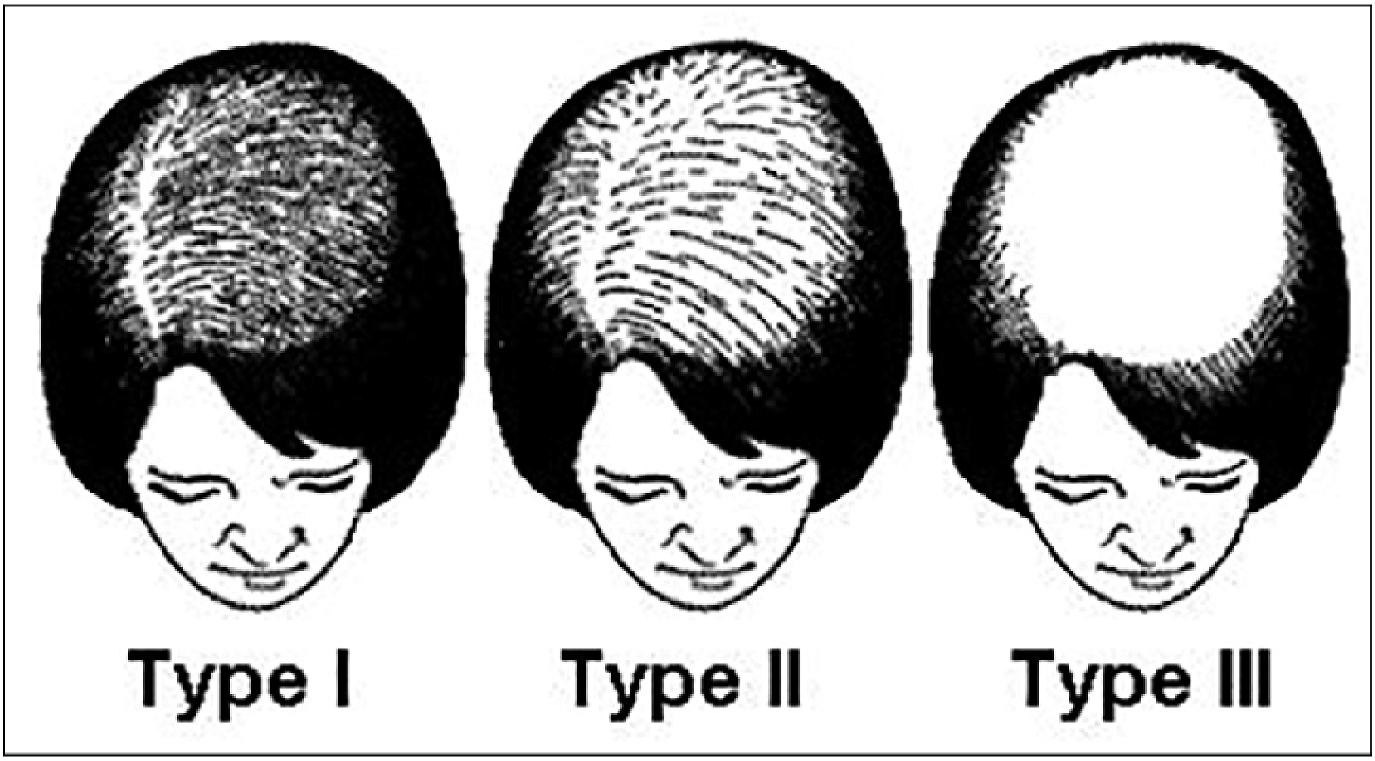

Wenn Sie die Schweregrade auf dem Bild betrachten, wo würden Sie sich einordnen?	◯ Typ 1<br>◯ Typ 2<br>◯ Typ 3
Leiden oder litten Sie je an übermässiger Körperbehaarung im Vergleich zu anderen Frauen? Betrachten Sie dazu das folgende Bild.	◯ Ja<br>◯ Nein<br>◯ Unbekannt

Damit Sie sich etwas unter "übermässiger Körperbehaarung" vorstellen können, sehen Sie dieses Bild mit verschiedenen Stadien der übermässigen Körperbehaarung an verschiedenen Körperstellen. Wenn eins davon auf Sie zutrifft, auch wenn nur an einer Körperstelle, beantworten Sie die Frage oben mit "JA".

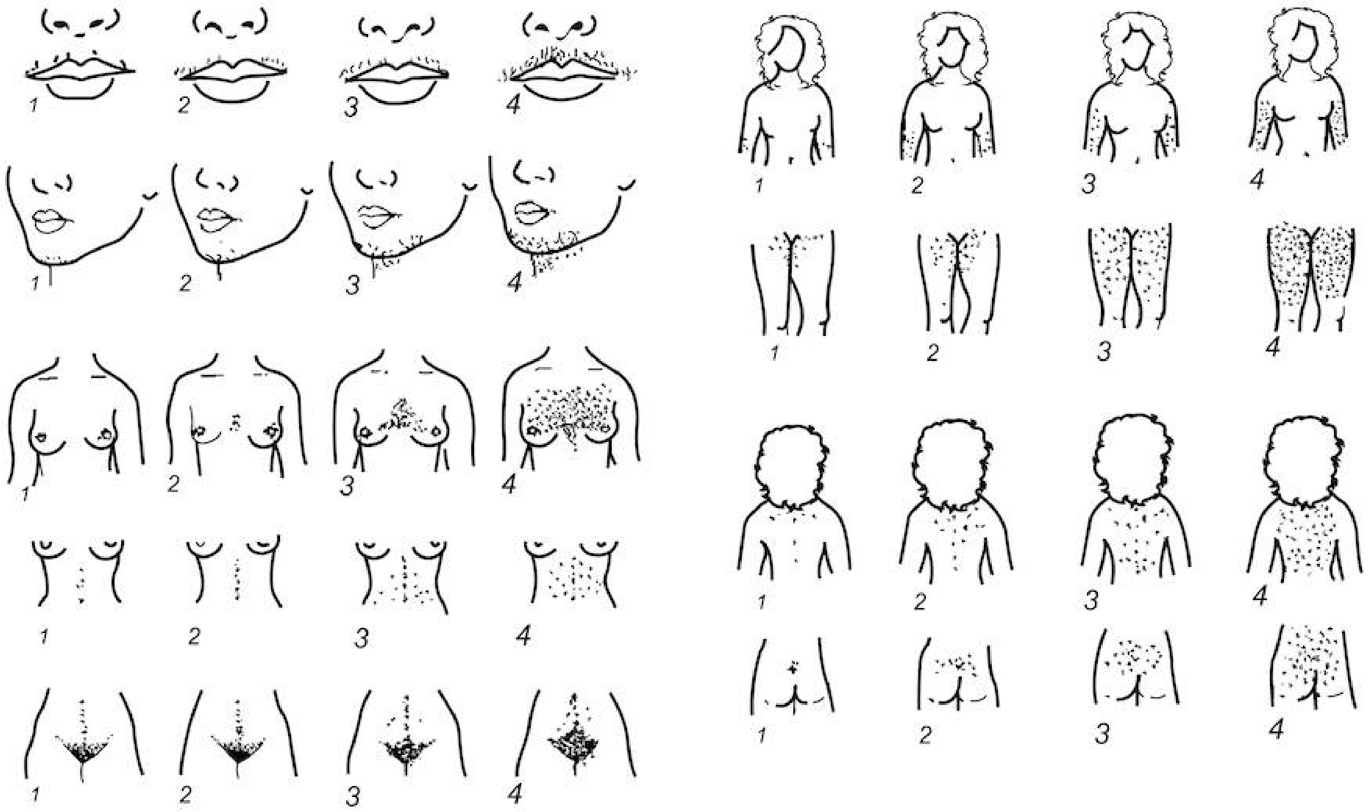

An welchen Körperstellen?

- ☐ Gesicht
- ☐ Brust
- ☐ Oberbauch (Brust bis Bauchnabel, wie bei Zeile 4 links)
- ☐ Unterbauch (Bauchnabel bis Intimbereich, wie bei Zeile 5 links)
- ☐ Oberarme
- ☐ Oberschenkel
- ☐ oberer Rücken (Nacken bis Taille, wie bei Zeile 3 rechts)
- ☐ unterer Rücken (Bereich über dem Po, wie bei Zeile 4 rechts)
- ☐ Andere

Körperstellen - Andere

____

(Bitte genauer beschreiben.)

Wo genau im Gesicht?

- ☐ Oberlippe
- ☐ Kinn
- ☐ Andere

Gesicht - Andere

____

(Bitte genauer beschreiben.)

Wenn Sie das Bild oben betrachten, wo würden Sie Ihre Behaarung an der Oberlippe einordnen?	◯ 1<br>◯ 2<br>◯ 3<br>◯ 4
Wenn Sie das Bild oben betrachten, wo würden Sie Ihre Behaarung am Kinn einordnen?	◯ 1<br>◯ 2<br>◯ 3<br>◯ 4
Wenn Sie das Bild oben betrachten, wo würden Sie Ihre andere Behaarung im Gesicht einordnen?	◯ 1<br>◯ 2<br>◯ 3<br>◯ 4
Wenn Sie das Bild oben betrachten, wo würden Sie Ihre Behaarung an der Brust einordnen?	◯ 1<br>◯ 2<br>◯ 3<br>◯ 4
Wenn Sie das Bild oben betrachten, wo würden Sie Ihre Behaarung am Oberbauch einordnen?	◯ 1<br>◯ 2<br>◯ 3<br>◯ 4
Wenn Sie das Bild oben betrachten, wo würden Sie Ihre Behaarung am Unterbauch einordnen?	◯ 1<br>◯ 2<br>◯ 3<br>◯ 4
Wenn Sie das Bild oben betrachten, wo würden Sie Ihre Behaarung an den Oberarmen einordnen?	◯ 1<br>◯ 2<br>◯ 3<br>◯ 4
Wenn Sie das Bild oben betrachten, wo würden Sie Ihre Behaarung an den Oberschenkeln einordnen?	◯ 1<br>◯ 2<br>◯ 3<br>◯ 4
Wenn Sie das Bild oben betrachten, wo würden Sie Ihre Behaarung am oberen Rücken einordnen?	◯ 1<br>◯ 2<br>◯ 3<br>◯ 4
Wenn Sie das Bild oben betrachten, wo würden Sie Ihre Behaarung am unteren Rücken einordnen?	◯ 1<br>◯ 2<br>◯ 3<br>◯ 4
Wenn Sie das Bild oben betrachten, wo würden Sie Ihre Behaarung an der "Anderen" Körperstelle (wie beschrieben) einordnen?	◯ 1<br>◯ 2<br>◯ 3<br>◯ 4

klinischer Hyperandrogenismus

Biochemischer Hyperandrogenismus

Wurden bei Ihnen schon einmal die männlichen und weiblichen Sexualhormone im Blut gemessen?	◯ Ja<br>◯ Nein<br>◯ Unbekannt
Wurde dabei ein erhöhter Anteil männlicher Hormone festgestellt?	◯ Ja<br>◯ Nein<br>◯ Unbekannt
Biochemischer Hyperandrogenismus	

Morphologisch Polyzystische Ovarien

Wurde bei Ihnen schon einmal ein Ultraschall über die Scheide durchgeführt?	◯ Ja<br>◯ Nein<br>◯ Unbekannt<br>(Dabei wird ein schmaler, stabförmiger Schallkopf in die Scheide eingeführt.)
Wurden dabei vergrösserte Eierstöcke festgestellt?	◯ Ja<br>◯ Nein<br>◯ Unbekannt
Wurden dabei eine erhöhte Anzahl Follikel (manchmal auch Zysten genannt) festgestellt?	◯ Ja<br>◯ Nein<br>◯ Unbekannt<br>(Dies sind "Bläschen" im Eierstock, die entstehen wenn sich unreife Eizellen im Eierstock sammeln.)
Morphologisch Polyzystische Ovarien	

Diagnose erhalten

Hat Ihr Frauenarzt/Ihre Frauenärztin Ihnen gesagt Sie haben ein Polyzystisches Ovarialsyndrom (PCOS)?	◯ Ja<br>◯ Nein<br>◯ Unbekannt

Differentialdiagnosen

Ist bei Ihnen ein Adrenogenitales Syndrom bekannt?	◯ Ja<br>◯ Nein / Unbekannt<br>(Falls Sie nicht wissen was das ist, kreuzen sie "Nein/Unbekannt" an.)
Ist bei Ihnen ein Prolaktinom bekannt?	◯ Ja<br>◯ Nein / Unbekannt<br>(Ein Prolaktinom ist ein gutartiger, Prolaktin produzierender Tumor der Hirnanhangdrüse.)
Differentialdiagnose erfüllt	

Leider erfüllen Sie die Kriterien zur Teilnahme an dieser Studie nicht. Wir danken Ihnen trotzdem für Ihre Bereitschaft und wünschen Ihnen alles Gute. Bitte klicken Sie auf "submit". Danach können Sie die Seite schliessen.

Einflussbereich 1 - Kosmetik

Bitte beantworten Sie die untenstehenden Fragen.

Herzlichen Dank!

Sie haben angegeben, dass Sie bisher noch keine Diagnose für PCOS erhalten haben. Gemäss unserer Befragung erfüllen Sie die Kriterien für PCOS. Bitte melden Sie sich bei Gelegenheit bei Ihrer Frauenärztin / Ihrem Frauenarzt für weitere Abklärungen.
Teilen Sie Ihr / Ihm mit, dass Sie bei einer Online-Studie zu den ESHRE-Guidelines bezüglich PCOS mitgemacht haben und dort die Kriterien erfüllt haben. Für Rückfragen stehe ich Ihnen oder Ihrem Frauenarzt gerne zur Verfügung (julia.estermann@students.unibe.ch)

Hat Ihr Aussehen sich Ihrer Meinung nach durch PCOS negativ verändert?
- ◯ Ja
- ◯ Nein

Bitte kreuzen Sie an, unter welchen kosmetischen Problemen Sie leiden oder litten.
- ☐ Akne und/oder fettige Haut
- ☐ Haarausfall
- ☐ vermehrte Körperbehaarung
- ☐ Übergewicht
- ☐ Andere
- ☐ keine

(Einige der Punkte wurden bereits früher in dieser Umfrage abgefragt. Bitte geben Sie diese trotzdem nochmals an. Es wird auf die hier angekreuzten Punkte noch genauer eingegangen.)

Welche anderen Probleme?

____
(Bitte genauer beschreiben.)

Akne und/oder fettige Haut

Sie haben früher in der Umfrage angegeben, dass Sie an Akne leiden/litten. Bitte beantworten Sie die folgenden Fragen dazu.

Stört/störte Sie die Akne und/oder fettige Haut?
- ◯ Ja
- ◯ Nein

Wurden Sie von Ihrer Frauenärztin / Ihrem Frauenarzt dazu beraten?
- ◯ Ja
- ◯ Nein

Wie zufrieden sind Sie mit der Beratung bezüglich Akne und/oder fettiger Haut?

0 ——— 50 ——— 100

(Place a mark on the scale above)

Hätten Sie sich bezüglich Akne und/oder fettiger Haut eine Beratung durch Ihre Frauenärztin / Ihren Frauenarzt gewünscht?
- ◯ Ja
- ◯ Nein

Haben Sie für Ihre Akne und/oder fettige Haut einen Therapieversuch gemacht?
- ◯ Ja
- ◯ Nein
- ◯ Unbekannt

Welche der folgenden Möglichkeiten haben Sie ausprobiert?	☐ Lebensstilveränderung (Ernährung, Bewegung, etc.)<br>☐ Medikamente (inkl. Pille)<br>☐ Psychotherapie<br>☐ bariatrische Operationen (z.B. Magen-Bypass)<br>☐ Andere<br>(Falls Sie bei einer Auswahlmöglichkeit nicht sicher sind, was damit gemeint ist, können Sie das Feld anklicken und die Unterkategorien anschauen. Falls nichts passendes dabei ist, können Sie das Feld auch wieder abwählen.)
Welche anderen Möglichkeiten?	____________________<br>(Bitte genauer beschreiben.)
Bitte kreuzen Sie die zutreffenden Lebensstilveränderungen an:	☐ Verhalten (z.B. Zielsetzung, Selbst-Dokumentation, Essgeschwindigkeit, etc.)<br>☐ Einstellung<br>☐ Ernährung (z.B. ausgewogener, weniger, etc.)<br>☐ Bewegung / körperliche Aktivität<br>☐ Gewicht (Gewichtsreduktion/-kontrolle)<br>☐ Andere
Welche anderen Lebensstilveränderungen?	____________________<br>(Bitte genauer beschreiben.)
Bitte kreuzen Sie die zutreffenden Medikamente an:	☐ Pille<br>☐ Antiandrogene<br>☐ Metformin<br>☐ Andere
Welche anderen Medikamente?	____________________<br>(Bitte genauer beschreiben.)
Waren die Massnahmen wirksam?	○ Ja, vollständig<br>○ Ja, teilweise<br>○ Nein, gar nicht
Welche der Massnahmen führen Sie aktuell noch durch?	☐ Lebensstilveränderung (Ernährung, Bewegung, etc.)<br>☐ Medikamente (inkl. Pille)<br>☐ Psychotherapie<br>☐ bariatrische Operationen (z.B. Magen-Bypass)<br>☐ Andere (wie oben beschrieben)<br>☐ Keine mehr
Bitte kreuzen Sie die zutreffenden Lebensstilveränderungen an, die sie aktuell noch durchführen:	☐ Verhalten (z.B. Zielsetzung, Selbst-Dokumentation, Essgeschwindigkeit, etc.)<br>☐ Einstellung<br>☐ Ernährung (z.B. ausgewogener, weniger, etc.)<br>☐ Bewegung / körperliche Aktivität<br>☐ Gewicht (Gewichtsreduktion/-kontrolle)<br>☐ Andere (wie oben beschrieben)

Bitte kreuzen Sie die zutreffenden Medikamente an, die sie aktuell noch nehmen:	☐ Pille<br>☐ Antiandrogene<br>☐ Metformin<br>☐ Andere (wie oben beschrieben)
Wurden Sie von Ihrer Frauenärztin / Ihrem Frauenarzt bei den Therapieversuchen betreut?	◯ Ja<br>◯ Nein
Wie zufrieden sind Sie mit der Betreuung der Therapie durch Ihre Frauenärztin / Ihren Frauenarzt?	0 50 100<br>*(Place a mark on the scale above)*
Hätten Sie sich bei der Therapie eine Betreuung durch Ihre Frauenärztin / Ihren Frauenarzt gewünscht?	◯ Ja<br>◯ Nein

Haarausfall

Sie haben früher in der Umfrage angegeben, dass Sie an übermässigem Haarausfall leiden/litten. Bitte beantworten Sie die folgenden Fragen dazu.

Stört/störte Sie der Haarausfall?	◯ Ja<br>◯ Nein
Wurden Sie von Ihrer Frauenärztin / Ihrem Frauenarzt dazu beraten?	◯ Ja<br>◯ Nein
Wie zufrieden sind Sie mit der Beratung bezüglich Haarausfall?	0 50 100<br>*(Place a mark on the scale above)*
Hätten Sie sich bezüglich Haarausfall eine Beratung durch Ihre Frauenärztin / Ihren Frauenarzt gewünscht?	◯ Ja<br>◯ Nein
Haben Sie für Ihren Haarausfall einen Therapieversuch gemacht?	◯ Ja<br>◯ Nein
Welche der folgenden Möglichkeiten haben Sie ausprobiert?	☐ Lebensstilveränderung (Ernährung, Bewegung, etc.)<br>☐ Medikamente (inkl. Pille)<br>☐ Psychotherapie<br>☐ bariatrische Operationen (z.B. Magen-Bypass)<br>☐ Andere<br>(Falls Sie bei einer Auswahlmöglichkeit nicht sicher sind, was damit gemeint ist, können Sie das Feld anklicken und die Unterkategorien anschauen. Falls nichts passendes dabei ist, können Sie das Feld auch wieder abwählen.)
Welche anderen Möglichkeiten?	____________________<br>(Bitte genauer beschreiben.)

Bitte kreuzen Sie die zutreffenden Lebensstilveränderungen an:

- ☐ Verhalten (z.B. Zielsetzung, Selbst-Dokumentation, Essgeschwindigkeit, etc.)
- ☐ Einstellung
- ☐ Ernährung (z.B. ausgewogener, weniger, etc.)
- ☐ Bewegung / körperliche Aktivität
- ☐ Gewicht (Gewichtsreduktion/-kontrolle)
- ☐ Andere

Welche anderen Lebensstilveränderungen?

____
(Bitte genauer beschreiben.)

Bitte kreuzen Sie die zutreffenden Medikamente an:

- ☐ Pille
- ☐ Antiandrogene
- ☐ Metformin
- ☐ Andere

Welche anderen Medikamente?

____
(Bitte genauer beschreiben.)

Waren die Massnahmen wirksam?

- ◯ Ja, vollständig
- ◯ Ja, teilweise
- ◯ Nein, gar nicht

Welche der Massnahmen führen Sie aktuell noch durch?

- ☐ Lebensstilveränderung (Ernährung, Bewegung, etc.)
- ☐ Medikamente (inkl. Pille)
- ☐ Psychotherapie
- ☐ bariatrische Operationen (z.B. Magen-Bypass)
- ☐ Andere (wie oben beschrieben)
- ☐ Keine mehr

Bitte kreuzen Sie die zutreffenden Lebensstilveränderungen an, die sie aktuell noch durchführen:

- ☐ Verhalten (z.B. Zielsetzung, Selbst-Dokumentation, Essgeschwindigkeit, etc.)
- ☐ Einstellung
- ☐ Ernährung (z.B. ausgewogener, weniger, etc.)
- ☐ Bewegung / körperliche Aktivität
- ☐ Gewicht (Gewichtsreduktion/-kontrolle)
- ☐ Andere (wie oben beschrieben)

Bitte kreuzen Sie die zutreffenden Medikamente an, die sie aktuell noch nehmen:

- ☐ Pille
- ☐ Antiandrogene
- ☐ Metformin
- ☐ Andere (wie oben beschrieben)

Wurden Sie von Ihrer Frauenärztin / Ihrem Frauenarzt bei den Therapieversuchen betreut?

- ◯ Ja
- ◯ Nein

Wie zufrieden sind Sie mit der Betreuung der Therapie durch Ihre Frauenärztin / Ihren Frauenarzt?

0 50 100

(Place a mark on the scale above)

Hätten Sie sich bei der Therapie eine Betreuung durch Ihre Frauenärztin / Ihren Frauenarzt gewünscht?

- ◯ Ja
- ◯ Nein

Körperbehaarung

Sie haben früher in der Umfrage angegeben, dass Sie an übermässiger Körperbehaarung leiden/litten. Bitte beantworten Sie die folgenden Fragen dazu.

Stört/störte Sie die vermehrte Körperbehaarung?

◯ Ja
◯ Nein

Wurden Sie von Ihrer Frauenärztin / Ihrem Frauenarzt dazu beraten?

◯ Ja
◯ Nein

Wie zufrieden sind Sie mit der Beratung bezüglich vermehrter Körperbehaarung?

0 50 100

(Place a mark on the scale above)

Hätten Sie sich bezüglich vermehrter Körperbehaarung eine Beratung durch Ihre Frauenärztin / Ihren Frauenarzt gewünscht?

◯ Ja
◯ Nein

Haben Sie für Ihre vermehrte Körperbehaarung einen Therapieversuch gemacht?

◯ Ja
◯ Nein

Welche der folgenden Möglichkeiten haben Sie ausprobiert?

☐ Lebensstilveränderung (Ernährung, Bewegung, etc.)
☐ Medikamente (inkl. Pille)
☐ Psychotherapie
☐ bariatrische Operationen (z.B. Magen-Bypass)
☐ Andere

(Falls Sie bei einer Auswahlmöglichkeit nicht sicher sind, was damit gemeint ist, können Sie das Feld anklicken und die Unterkategorien anschauen. Falls nichts passendes dabei ist, können Sie das Feld auch wieder abwählen.)

Welche anderen Möglichkeiten?

____
(Bitte genauer beschreiben.)

Bitte kreuzen Sie die zutreffenden Lebensstilveränderungen an:

☐ Verhalten (z.B. Zielsetzung, Selbst-Dokumentation, Essgeschwindigkeit, etc.)
☐ Einstellung
☐ Ernährung (z.B. ausgewogener, weniger, etc.)
☐ Bewegung / körperliche Aktivität
☐ Gewicht (Gewichtsreduktion/-kontrolle)
☐ Andere

Welche anderen Lebensstilveränderungen?

____
(Bitte genauer beschreiben.)

Bitte kreuzen Sie die zutreffenden Medikamente an:

☐ Pille
☐ Antiandrogene
☐ Metformin
☐ Andere

Welche anderen Medikamente?	______________________ (Bitte genauer beschreiben.)
Waren die Massnahmen wirksam?	◯ Ja, vollständig<br>◯ Ja, teilweise<br>◯ Nein, gar nicht
Welche der Massnahmen führen Sie aktuell noch durch?	☐ Lebensstilveränderung (Ernährung, Bewegung, etc.)<br>☐ Medikamente (inkl. Pille)<br>☐ Psychotherapie<br>☐ bariatrische Operationen (z.B. Magen-Bypass)<br>☐ Andere (wie oben beschrieben)<br>☐ Keine mehr
Bitte kreuzen Sie die zutreffenden Lebensstilveränderungen an, die sie aktuell noch durchführen:	☐ Verhalten (z.B. Zielsetzung, Selbst-Dokumentation, Essgeschwindigkeit, etc.)<br>☐ Einstellung<br>☐ Ernährung (z.B. ausgewogener, weniger, etc.)<br>☐ Bewegung / körperliche Aktivität<br>☐ Gewicht (Gewichtsreduktion/-kontrolle)<br>☐ Andere (wie oben beschrieben)
Bitte kreuzen Sie die zutreffenden Medikamente an, die sie aktuell noch nehmen:	☐ Pille<br>☐ Antiandrogene<br>☐ Metformin<br>☐ Andere (wie oben beschrieben)
Wurden Sie von Ihrer Frauenärztin / Ihrem Frauenarzt bei den Therapieversuchen betreut?	◯ Ja<br>◯ Nein
Wie zufrieden sind Sie mit der Betreuung der Therapie durch Ihre Frauenärztin / Ihren Frauenarzt?	0 50 100<br>*(Place a mark on the scale above)*
Hätten Sie sich bei der Therapie eine Betreuung durch Ihre Frauenärztin / Ihren Frauenarzt gewünscht?	◯ Ja<br>◯ Nein

Übergewicht

Gemäss der Berechnung Ihres BMIs leiden Sie an leichtem Übergewicht. Bitte beantworten Sie die folgenden Fragen dazu.

Gemäss der Berechnung Ihres BMIs leiden Sie an mässigem Übergewicht. Bitte beantworten Sie die folgenden Fragen dazu.

Gemäss der Berechnung Ihres BMIs leiden Sie an starkem Übergewicht. Bitte beantworten Sie die folgenden Fragen dazu.

Stört/störte Sie das Übergewicht?	◯ Ja<br>◯ Nein
Wurden Sie von Ihrer Frauenärztin / Ihrem Frauenarzt dazu beraten?	◯ Ja<br>◯ Nein

Wie zufrieden sind Sie mit der Beratung bezüglich Übergewicht?	0 50 100 (Place a mark on the scale above)
Hätten Sie sich bezüglich Übergewicht eine Beratung durch Ihre Frauenärztin / Ihren Frauenarzt gewünscht?	◯ Ja<br>◯ Nein
Haben Sie für Ihr Übergewicht einen Therapieversuch gemacht?	◯ Ja<br>◯ Nein
Welche der folgenden Möglichkeiten haben Sie ausprobiert?	☐ Lebensstilveränderung (Ernährung, Bewegung, etc.)<br>☐ Medikamente (inkl. Pille)<br>☐ Psychotherapie<br>☐ bariatrische Operationen (z.B. Magen-Bypass)<br>☐ Andere<br>(Falls Sie bei einer Auswahlmöglichkeit nicht sicher sind, was damit gemeint ist, können Sie das Feld anklicken und die Unterkategorien anschauen. Falls nichts passendes dabei ist, können Sie das Feld auch wieder abwählen.)
Welche anderen Möglichkeiten?	____________________<br>(Bitte genauer beschreiben.)
Bitte kreuzen Sie die zutreffenden Lebensstilveränderungen an:	☐ Verhalten (z.B. Zielsetzung, Selbst-Dokumentation, Essgeschwindigkeit, etc.)<br>☐ Einstellung<br>☐ Ernährung (z.B. ausgewogener, weniger, etc.)<br>☐ Bewegung / körperliche Aktivität<br>☐ Gewicht (Gewichtsreduktion/-kontrolle)<br>☐ Andere
Welche anderen Lebensstilveränderungen?	____________________<br>(Bitte genauer beschreiben.)
Bitte kreuzen Sie die zutreffenden Medikamente an:	☐ Pille<br>☐ Antiandrogene<br>☐ Metformin<br>☐ Andere
Welche anderen Medikamente?	____________________<br>(Bitte genauer beschreiben.)
Waren die Massnahmen wirksam?	◯ Ja, vollständig<br>◯ Ja, teilweise<br>◯ Nein, gar nicht

Welche der Massnahmen führen Sie aktuell noch durch?	☐ Lebensstilveränderung (Ernährung, Bewegung, etc.)<br>☐ Medikamente (inkl. Pille)<br>☐ Psychotherapie<br>☐ bariatrische Operationen (z.B. Magen-Bypass)<br>☐ Andere (wie oben beschrieben)<br>☐ Keine mehr
Bitte kreuzen Sie die zutreffenden Lebensstilveränderungen an, die sie aktuell noch durchführen:	☐ Verhalten (z.B. Zielsetzung, Selbst-Dokumentation, Essgeschwindigkeit, etc.)<br>☐ Einstellung<br>☐ Ernährung (z.B. ausgewogener, weniger, etc.)<br>☐ Bewegung / körperliche Aktivität<br>☐ Gewicht (Gewichtsreduktion/-kontrolle)<br>☐ Andere (wie oben beschrieben)
Bitte kreuzen Sie die zutreffenden Medikamente an, die sie aktuell noch nehmen:	☐ Pille<br>☐ Antiandrogene<br>☐ Metformin<br>☐ Andere (wie oben beschrieben)
Wurden Sie von Ihrer Frauenärztin / Ihrem Frauenarzt bei den Therapieversuchen betreut?	◯ Ja<br>◯ Nein
Wie zufrieden sind Sie mit der Betreuung der Therapie durch Ihre Frauenärztin / Ihren Frauenarzt?	0 50 100<br>*(Place a mark on the scale above)*
Hätten Sie sich bei der Therapie eine Betreuung durch Ihre Frauenärztin / Ihren Frauenarzt gewünscht?	◯ Ja<br>◯ Nein

Andere

Sie haben angekreuzt, dass sie unter einem anderen kosmetischen Problem leiden/litten. Sie haben dieses Problem oben im Textfeld beschrieben. Stört/störte Sie dieses Problem?	◯ Ja<br>◯ Nein
Wurden Sie von Ihrer Frauenärztin / Ihrem Frauenarzt dazu beraten?	◯ Ja<br>◯ Nein
Wie zufrieden sind Sie mit der Beratung bezüglich dieses anderen Problemes?	0 50 100<br>*(Place a mark on the scale above)*
Hätten Sie sich bezüglich dieses anderen Problemes eine Beratung durch Ihre Frauenärztin / Ihren Frauenarzt gewünscht?	◯ Ja<br>◯ Nein
Haben Sie für dieses Problem einen Therapieversuch gemacht?	◯ Ja<br>◯ Nein

Welche der folgenden Möglichkeiten haben Sie ausprobiert?	☐ Lebensstilveränderung (Ernährung, Bewegung, etc.)<br>☐ Medikamente (inkl. Pille)<br>☐ Psychotherapie<br>☐ bariatrische Operationen (z.B. Magen-Bypass)<br>☐ Andere<br>(Falls Sie bei einer Auswahlmöglichkeit nicht sicher sind, was damit gemeint ist, können Sie das Feld anklicken und die Unterkategorien anschauen. Falls nichts passendes dabei ist, können Sie das Feld auch wieder abwählen.)
Welche anderen Möglichkeiten?	__________________________<br>(Bitte genauer beschreiben.)
Bitte kreuzen Sie die zutreffenden Lebensstilveränderungen an:	☐ Verhalten (z.B. Zielsetzung, Selbst-Dokumentation, Essgeschwindigkeit, etc.)<br>☐ Einstellung<br>☐ Ernährung (z.B. ausgewogener, weniger, etc.)<br>☐ Bewegung / körperliche Aktivität<br>☐ Gewicht (Gewichtsreduktion/-kontrolle)<br>☐ Andere
Welche anderen Lebensstilveränderungen?	__________________________<br>(Bitte genauer beschreiben.)
Bitte kreuzen Sie die zutreffenden Medikamente an:	☐ Pille<br>☐ Antiandrogene<br>☐ Metformin<br>☐ Andere
Welche anderen Medikamente?	__________________________<br>(Bitte genauer beschreiben.)
Waren die Massnahmen wirksam?	◯ Ja, vollständig<br>◯ Ja, teilweise<br>◯ Nein, gar nicht
Welche der Massnahmen führen Sie aktuell noch durch?	☐ Lebensstilveränderung (Ernährung, Bewegung, etc.)<br>☐ Medikamente (inkl. Pille)<br>☐ Psychotherapie<br>☐ bariatrische Operationen (z.B. Magen-Bypass)<br>☐ Andere (wie oben beschrieben)<br>☐ Keine mehr
Bitte kreuzen Sie die zutreffenden Lebensstilveränderungen an, die sie aktuell noch durchführen:	☐ Verhalten (z.B. Zielsetzung, Selbst-Dokumentation, Essgeschwindigkeit, etc.)<br>☐ Einstellung<br>☐ Ernährung (z.B. ausgewogener, weniger, etc.)<br>☐ Bewegung / körperliche Aktivität<br>☐ Gewicht (Gewichtsreduktion/-kontrolle)<br>☐ Andere (wie oben beschrieben)

Bitte kreuzen Sie die zutreffenden Medikamente an, die sie aktuell noch nehmen:	☐ Pille<br>☐ Antiandrogene<br>☐ Metformin<br>☐ Andere (wie oben beschrieben)
Wurden Sie von Ihrer Frauenärztin / Ihrem Frauenarzt bei den Therapieversuchen betreut?	◯ Ja<br>◯ Nein
Wie zufrieden sind Sie mit der Betreuung der Therapie durch Ihre Frauenärztin / Ihren Frauenarzt?	0 50 100<br>*(Place a mark on the scale above)*
Hätten Sie sich bei der Therapie eine Betreuung durch Ihre Frauenärztin / Ihren Frauenarzt gewünscht?	◯ Ja<br>◯ Nein

Gesamtzufriedenheit Kosmetik

Wie zufrieden sind Sie gesamthaft mit der Betreuung durch Ihre Frauenärztin / Ihren Frauenarzt bezüglich Kosmetik?	0 50 100<br>*(Place a mark on the scale above)*
Würden Sie sich zusätzliche Beratung wünschen?	◯ Ja<br>◯ Nein
Bitte kreuzen Sie an, was Sie sich zusätzlich wünschen würden:	☐ mehr Beratung / Beruhigung<br>☐ mehr Abgabe von Informationen (z.B. Broschüren)<br>☐ mehr Möglichkeiten Fragen zu stellen<br>☐ mehr Tests (Bluttests, Ultraschall, etc.)<br>☐ mehr Therapie-Möglichkeiten<br>☐ Andere
Welche andere zusätzliche Beratung?	____________________<br>(Bitte genauer beschreiben.)

Einflussbereich 2 - Stoffwechsel

Bitte beantworten Sie die untenstehenden Fragen.

Herzlichen Dank!

Ist bei Ihnen eine der folgenden Erkrankungen bekannt? Bitte kreuzen Sie die Zutreffenden an.	☐ Diabetes mellitus<br>☐ Schwangerschaftsdiabetes (aktuell oder in der Vergangenheit)<br>☐ Diabetes-Vorstufe (erhöhter Blutzucker, aber noch kein Diabetes)<br>☐ Keine der genannten
Welcher Typ Diabetes?	○ Typ 1<br>○ Typ 2
Ist dieser Diabetes nach der Schwangerschaft wieder verschwunden?	○ Ja<br>○ Nein
Wurde bei Ihnen schon einmal ein Blutzucker-Test durchgeführt?	○ Ja<br>○ Nein<br>○ Unbekannt<br>(Dazu gehörten nüchtern Blutzucker-Messung, Langzeitblutzuckermessung (HbA1c) oder Zuckerbelastungstest (=oGGT, Zuckerlösung trinken, danach mehrmals Blutzucker messen))
Wurden Sie von Ihrer Frauenärztin / Ihrem Frauenarzt bezüglich Ihrer Zucker-Erkrankung beraten?	○ Ja<br>○ Nein
Wie zufrieden sind Sie mit der Beratung bezüglich Ihrer Zucker-Erkrankung?	0 50 100<br>*(Place a mark on the scale above)*
Wurden Sie von Ihrem Hausarzt/-ärztin oder Ihrem Diabetologen bezüglich Ihrer Zucker-Erkrankung beraten?	○ Ja<br>○ Nein
Hätten Sie sich bezüglich Ihrer Zuckererkrankung eine Beratung durch Ihre Frauenärztin / Ihren Frauenarzt gewünscht?	○ Ja<br>○ Nein

Therapie Zuckererkrankung

Haben Sie für Ihre Zucker-Erkrankung eine Therapie oder einen Therapieversuch gemacht?	○ Ja<br>○ Nein<br>○ Unbekannt

Welche der folgenden Möglichkeiten haben Sie ausprobiert?	☐ Lebensstilveränderung (Ernährung, Bewegung, etc.)<br>☐ Medikamente<br>☐ Psychotherapie<br>☐ bariatrische Operationen (z.B. Magen-Bypass)<br>☐ Andere<br>(Falls Sie bei einer Auswahlmöglichkeit nicht sicher sind, was damit gemeint ist, können Sie das Feld anklicken und die Unterkategorien anschauen. Falls nichts passendes dabei ist, können Sie das Feld auch wieder abwählen.)
Welche anderen Möglichkeiten?	________________________<br>(Bitte genauer beschreiben.)
Bitte kreuzen Sie die zutreffenden Lebensstilveränderungen an:	☐ Verhalten (z.B. Zielsetzung, Selbst-Dokumentation, Essgeschwindigkeit, etc.)<br>☐ Einstellung<br>☐ Ernährung (z.B. ausgewogener, weniger, etc.)<br>☐ Bewegung / körperliche Aktivität<br>☐ Gewicht (Gewichtsreduktion/-kontrolle)<br>☐ Andere
Welche anderen Lebensstilveränderungen?	________________________<br>(Bitte genauer beschreiben.)
Bitte kreuzen Sie die zutreffenden Medikamente an:	☐ Metformin<br>☐ andere Diabetes-Tabletten<br>☐ Insulin (Spritzen/Pen)<br>☐ Andere
Welche anderen Medikamente?	________________________<br>(Bitte genauer beschreiben.)
Waren die Massnahmen wirksam?	○ Ja, vollständig<br>○ Ja, teilweise<br>○ Nein, gar nicht
Welche der Massnahmen führen Sie aktuell noch durch?	☐ Lebensstilveränderung (Ernährung, Bewegung, etc.)<br>☐ Medikamente<br>☐ Psychotherapie<br>☐ bariatrische Operationen (z.B. Magen-Bypass)<br>☐ Andere (wie oben beschrieben)<br>☐ Keine mehr
Bitte kreuzen Sie die zutreffenden Lebensstilveränderungen an, die sie aktuell noch durchführen:	☐ Verhalten (z.B. Zielsetzung, Selbst-Dokumentation, Essgeschwindigkeit, etc.)<br>☐ Einstellung<br>☐ Ernährung (z.B. ausgewogener, weniger, etc.)<br>☐ Bewegung / körperliche Aktivität<br>☐ Gewicht (Gewichtsreduktion/-kontrolle)<br>☐ Andere (wie oben beschrieben)

Bitte kreuzen Sie die zutreffenden Medikamente an, die sie aktuell noch nehmen:	☐ Metformin<br>☐ andere Diabetes-Tabletten<br>☐ Insulin (Spritzen/Pen)<br>☐ Andere (wie oben beschrieben)
Wurden Sie von Ihrer Frauenärztin / Ihrem Frauenarzt bei den Therapieversuchen betreut?	◯ Ja<br>◯ Nein
Wie zufrieden sind Sie mit der Betreuung der Therapie durch Ihre Frauenärztin / Ihren Frauenarzt?	0 50 100<br>*(Place a mark on the scale above)*
Hätten Sie sich bei der Therapie eine Betreuung durch Ihre Frauenärztin / Ihren Frauenarzt gewünscht?	◯ Ja<br>◯ Nein

Gesamtzufriedenheit Stoffwechsel / Zuckerkrankheiten

Wie zufrieden sind Sie gesamthaft mit der Betreuung durch Ihre Frauenärztin / Ihren Frauenarzt bezüglich Zucker-Erkrankungen?	0 50 100<br>*(Place a mark on the scale above)*
Würden Sie sich zusätzliche Beratung wünschen?	◯ Ja<br>◯ Nein
Bitte kreuzen Sie an, was Sie sich zusätzlich wünschen würden:	☐ mehr Beratung / Beruhigung<br>☐ mehr Abgabe von Informationen (z.B. Broschüren)<br>☐ mehr Möglichkeiten Fragen zu stellen<br>☐ mehr Tests (Bluttests, Ultraschall, etc.)<br>☐ mehr Therapie-Möglichkeiten<br>☐ Andere
Welche andere zusätzliche Beratung?	____________________<br>(Bitte genauer beschreiben.)

Einflussbereich 3 - Endometrium

Bitte beantworten Sie die untenstehenden Fragen.

Herzlichen Dank!

Aktuell unregelmässige Periode

Sie haben weiter oben angekreuzt, dass Ihre Periode aktuell nicht regelmässig ist. Stört Sie diese Unregelmässigkeit?	◯ Ja<br>◯ Nein
Wurden Sie von Ihrer Frauenärztin zu Ihrer aktuell unregelmässigen Periode beraten?	◯ Ja<br>◯ Nein
Wie zufrieden sind Sie mit der Beratung bezüglich Ihrer aktuell unregelmässigen Periode?	0 50 100<br>*(Place a mark on the scale above)*
Hätten Sie sich bezüglich Ihrer aktuell unregelmässigen Periode eine Beratung durch Ihre Frauenärztin / Ihren Frauenarzt gewünscht?	◯ Ja<br>◯ Nein

Früher unregelmässige Periode

Sie haben weiter oben angekreuzt, dass Ihre Periode früher nicht regelmässig war. Störte Sie diese Unregelmässigkeit?	◯ Ja<br>◯ Nein
Wurden Sie damals von Ihrer Frauenärztin zu Ihrer unregelmässigen Periode beraten?	◯ Ja<br>◯ Nein
Wie zufrieden sind Sie mit der damaligen Beratung bezüglich Ihrer unregelmässigen Periode?	0 50 100<br>*(Place a mark on the scale above)*
Hätten Sie sich damals bezüglich Ihrer unregelmässigen Periode eine Beratung durch Ihre Frauenärztin / Ihren Frauenarzt gewünscht?	◯ Ja<br>◯ Nein

Pille

Hat Ihre Frauenärztin / Ihr Frauenarzt Sie bezüglich Möglichkeit einer Pille zur Behandlung der unregelmässigen Periode beraten?	◯ Ja<br>◯ Nein<br>(Hier geht es nur darum, ob die Pille jemals thematisiert wurde. Dies ist unabhängig davon, ob diese dann auch gewünscht und genommen wurde.)
Haben Sie die Pille dann auch genommen?	◯ Ja<br>◯ Nein
Haben Sie die Pille (auch) aus anderen Gründen genommen?	◯ Ja<br>◯ Nein

Aus welchen Gründen?	☐ Akne und/oder fettige Haut<br>☐ Haarausfall<br>☐ vermehrte Körperbehaarung<br>☐ Verhütung<br>☐ Andere
Welche anderen Gründe?	__________________<br>(Bitte genauer beschreiben.)
Hat die Pille gegen die unregelmässige Periode geholfen?	◯ Ja, vollständig<br>◯ Ja, teilweise<br>◯ Nein, gar nicht
Hat die Pille gegen die anderen Probleme geholfen?	◯ Ja, vollständig<br>◯ Ja, teilweise<br>◯ Nein, gar nicht
Nehmen Sie die Pille aktuell noch?	◯ Ja<br>◯ Nein
Weshalb haben Sie die Pille gestoppt?	☐ Sie half zu wenig gegen die Probleme<br>☐ Ich möchte keine Hormone mehr nehmen<br>☐ Kinderwunsch<br>☐ Nebenwirkungen<br>☐ Andere
Welche anderen Gründe?	__________________<br>(Bitte genauer beschreiben.)
Weshalb haben Sie sich gegen die Pille entschieden?	☐ Ich möchte keine Hormone nehmen<br>☐ Kinderwunsch<br>☐ Angst vor Nebenwirkungen<br>☐ Andere
Welche anderen Gründe?	__________________<br>(Bitte genauer beschreiben.)
Wie zufrieden sind Sie mit der Beratung bezüglich Pille durch Ihre Frauenärztin / Ihren Frauenarzt?	0 50 100<br>*(Place a mark on the scale above)*
Hätten Sie sich bezüglich Pille eine Beratung durch Ihre Frauenärztin / Ihren Frauenarzt gewünscht?	◯ Ja<br>◯ Nein

Gesamtzufriedenheit Endometrium / Zyklus

Wie zufrieden sind Sie gesamthaft mit der Betreuung durch Ihre Frauenärztin / Ihren Frauenarzt bezüglich Menstruationszyklus?

0 50 100

(Place a mark on the scale above)

Würden Sie sich zusätzliche Beratung wünschen?

- ◯ Ja
- ◯ Nein

Bitte kreuzen Sie an, was Sie sich zusätzlich wünschen würden:

- ☐ mehr Beratung / Beruhigung
- ☐ mehr Abgabe von Informationen (z.B. Broschüren)
- ☐ mehr Möglichkeiten Fragen zu stellen
- ☐ mehr Tests (Bluttests, Ultraschall, etc.)
- ☐ mehr Therapie-Möglichkeiten
- ☐ Andere

Welche andere zusätzliche Beratung?

____
(Bitte genauer beschreiben.)

Einflussbereich 4 - Kinderwunsch

Bitte beantworten Sie die untenstehenden Fragen.

Herzlichen Dank!

Kinderwunsch

Wünschen Sie sich irgendwann einmal Kinder?

◯ Ja
◯ Nein
◯ Unbekannt

Wünschen Sie sich aktuell Kinder?

◯ Ja
◯ Nein
(Gemeint ist der Wunsch, möglichst bald Kinder zu bekommen. Dazu müssen noch keine Versuche stattgefunden haben schwanger zu werden.)

Ungefähr wie lange haben Sie schon einen aktuellen Kinderwunsch?

◯ bis 1 Monat
◯ 1-3 Monate
◯ 3-6 Monate
◯ 6-9 Monate
◯ 9-12 Monate
◯ 1-2 Jahre
◯ 2-3 Jahre
◯ 3-4 Jahre
◯ über 4 Jahre
◯ Unbekannt

In ungefähr wie vielen Jahren wünschen Sie sich Kinder?

◯ 1 ◯ 2 ◯ 3 ◯ 4
◯ 5 ◯ 6 ◯ 7 ◯ 8
◯ 9 ◯ 10 ◯ über 10
◯ Unbekannt

Wurden Sie von Ihrer Frauenärztin / Ihrem Frauenarzt bezüglich Kinderwunsch beraten?

◯ Ja
◯ Nein
(Also über die möglichen Schwierigkeiten und die Möglichkeiten aufgeklärt.)

Wie zufrieden sind Sie mit der Beratung bezüglich Kinderwunsch?

0 50 100

(Place a mark on the scale above)

Hätten Sie sich eine Beratung bezüglich Kinderwunsch gewünscht?

◯ Ja
◯ Nein

Schwangerschaft

Waren Sie schon einmal schwanger?

◯ Ja
◯ Nein

Wie oft waren Sie schon schwanger?

____
(Inklusive Fehlgeburten und/oder gewünschte Abbrüche. Bitte schreiben Sie die Anzahl Schwangerschaften ins Feld (z.B.: 3).)

Hatten Sie schon einmal eine Fehlgeburt?	◯ Ja<br>◯ Nein
Wie viele Schwangerschaften endeten in einer Fehlgeburt?	____________________<br>(Bitte schreiben Sie eine Zahl.)
Wie weit war die Schwangerschaft fortgeschritten, als Sie eine Fehlgeburt erlitten.	☐ < 12. Schwangerschaftswoche (Frühabort)<br>☐ 12. - 22. Schwangerschaftswoche (Spätabort)<br>☐ > 22.Schwangerschaftswoche und/oder Gewicht >500g (Totgeburt)<br>(Falls Sie mehrere Fehlgeburten hatten, können Sie auch mehrere Felder anwählen.)
Hatten Sie schon einmal einen gewollten Schwangerschaftsabbruch?	◯ Ja<br>◯ Nein
Wie viele Schwangerschaften endeten in einem gewollten Abbruch?	____________________<br>(Bitte schreiben Sie eine Zahl.)
Haben Sie schon einmal über 1 Jahr erfolglos versucht schwanger zu werden?	◯ Ja<br>◯ Nein<br>(Versuch schwanger zu werden = regelmässiger, ungeschützter Geschlechtsverkehr zum richtigen Zeitpunkt)
Versuchen Sie aktuell seit über einem Jahr schwanger zu werden?	◯ Ja<br>◯ Nein
Wie lange versuchen Sie schon schwanger zu werden?	◯ 12 Monate<br>◯ 12-18 Monate<br>◯ 18-24 Monate<br>◯ 24-36 Monate<br>◯ 36-48 Monate<br>◯ über 4 Jahre
Wie lange ist es her, dass sie über ein Jahr erfolglos versuchten schwanger zu werden? [Jahre]	____________________<br>(Bitte geben Sie die Zeit in ganzen Jahren an (z.B.: 4))
Wie lange habe Sie damals erfolglos versucht schwanger zu werden?	◯ 12 Monate<br>◯ 12-18 Monate<br>◯ 18-24 Monate<br>◯ 24-36 Monate<br>◯ 36-48 Monate<br>◯ über 4 Jahre
Wurden Sie von Ihrer Frauenärztin / Ihrem Frauenarzt bezüglich der erfolglosen Schwangerschaftsversuche beraten?	◯ Ja<br>◯ Nein<br>(Also über die möglichen Gründe und die Möglichkeiten aufgeklärt.)
Wie zufrieden sind Sie mit der Beratung bezüglich der erfolglosen Schwangerschaftsversuche?	0 — 50 — 100<br>*(Place a mark on the scale above)*

Hätten Sie sich bezüglich der erfolglosen Schwangerschaftsversuche eine Betreuung durch Ihre Frauenärztin / Ihren Frauenarzt gewünscht?	◯ Ja<br>◯ Nein
Haben Sie schon einmal eine Therapie beansprucht um schwanger zu werden?	◯ Ja<br>◯ Nein<br>(Falls Sie nicht sicher sind, was damit gemeint ist, können Sie "Ja" anklicken und die Unterkategorien anschauen. Falls nichts Passendes dabei ist, können Sie auch wieder auf "Nein" wechseln.)
Welche der folgenden Möglichkeiten haben Sie ausprobiert?	☐ Lebensstilveränderungen<br>☐ Medikamente (Tabletten oder Spritzen zur Stimulation der Eizellreifung)<br>☐ Operationen<br>☐ In-vitro Befruchtung (im Labor befruchtet)<br>☐ Andere<br>(Falls Sie bei einer Auswahlmöglichkeit nicht sicher sind, was damit gemeint ist, können Sie das Feld anklicken und die Unterkategorien anschauen. Falls nichts passendes dabei ist, können Sie das Feld auch wieder abwählen.)
Welche anderen Möglichkeiten?	____________________________________<br>(Bitte genauer beschreiben.)
Bitte kreuzen Sie die zutreffenden Lebensstilveränderungen an:	☐ Verhalten (z.B. Zielsetzung, Selbst-Dokumentation, etc.)<br>☐ Einstellung<br>☐ Ernährung (z.B. ausgewogener, weniger, etc.)<br>☐ Bewegung / körperliche Aktivität<br>☐ Gewicht (Gewichtsreduktion/-kontrolle)<br>☐ Blutzucker-Optimierung<br>☐ Blutdruck-Optimierung<br>☐ Verzicht auf Rauchen, Alkohol und andere Drogen<br>☐ Schlaf-Optimierung<br>☐ Veränderungen bei der Sexualität<br>☐ Andere
Welche anderen Lebensstilveränderungen?	____________________________________<br>(Bitte genauer beschreiben.)
Bitte kreuzen Sie die zutreffenden Medikamente an:	☐ Letrozol (Tablette, ="Femara")<br>☐ Clomifen (Tablette, ="Serophene" oder "Clomid")<br>☐ Metformin (Tablette)<br>☐ Gonadotropine (Spritzen, z.B. "Choriomon", "Menopur", "Merional", "Fostimon", "Gonal-F", etc.)<br>☐ Andere<br>☐ Unbekannt<br>(Falls Sie nicht mehr wissen, welche Medikamente Sie erhalten haben, kreuzen Sie "Unbekannt" an.)

Welche anderen Medikamente?	__________________________________<br>(Bitte genauer beschreiben.)
Bitte kreuzen Sie die zutreffenden Operationen an:	☐ Operation an den Eierstöcken<br>☐ Prüfung der Durchgängigkeit der Eileiter (=Chromopertubation/Blauprobe)<br>☐ bariatrische Operationen (z.B. Magen-Bypass)<br>☐ Andere<br>☐ Unbekannt<br>(Falls Sie nicht mehr wissen, was operiert wurde, kreuzen Sie "Unbekannt" an.)
Welche anderen Operationen?	__________________________________<br>(Bitte genauer beschreiben.)
Wurden Sie mit Hilfe einer dieser Therapien schwanger?	◯ Ja<br>◯ Nein
Haben Sie Kinder, die mit der Hilfe einer solchen Therapie entstanden?	◯ Ja<br>◯ Nein
Wie viele Ihrer Kinder sind mit der Hilfe einer solchen Therapie entstanden?	◯ Alle<br>◯ 1<br>◯ 2<br>◯ 3<br>◯ 4<br>◯ 5<br>◯ 6<br>◯ 7<br>◯ 8<br>◯ 9<br>◯ 10<br>◯ über 10
Führen Sie eine der Therapien aktuell gerade durch oder ist bereits etwas davon geplant?	◯ Ja<br>◯ Nein
Welche der folgenden Möglichkeiten führen Sie aktuell durch oder sind bald geplant?	☐ Lebensstilveränderungen<br>☐ Medikamente (Tabletten oder Spritzen zur Stimulation der Eizellreifung)<br>☐ Operationen<br>☐ In-vitro Befruchtung (im Labor befruchtet)<br>☐ Andere

Bitte kreuzen Sie die zutreffenden Lebensstilveränderungen an, die Sie aktuell noch durchführen:	☐ Verhalten (z.B. Zielsetzung, Selbst-Dokumentation, etc.)<br>☐ Einstellung<br>☐ Ernährung (z.B. ausgewogener, weniger, etc.)<br>☐ Bewegung / körperliche Aktivität<br>☐ Gewicht (Gewichtsreduktion/-kontrolle)<br>☐ Blutzucker-Optimierung<br>☐ Blutdruck-Optimierung<br>☐ Verzicht auf Rauchen, Alkohol und andere Drogen<br>☐ Schlaf-Optimierung<br>☐ Veränderungen bei der Sexualität<br>☐ Andere (wie oben beschrieben)
Bitte kreuzen Sie die zutreffenden Medikamente an, die Sie aktuell noch nehmen:	☐ Letrozol (Tablette, ="Femara")<br>☐ Clomifen (Tablette, ="Serophene" oder "Clomid")<br>☐ Metformin (Tablette)<br>☐ Gonadotropine (Spritzen, z.B. "Choriomon", "Menopur", "Merional", "Fostimon", "Gonal-F", etc.)<br>☐ Andere (wie oben beschrieben)<br>☐ Unbekannt<br>(Falls Sie nicht mehr wissen, wie das Medikament heisst, kreuzen Sie "Unbekannt" an.)
Bitte kreuzen Sie die zutreffenden Operationen an, die aktuell geplant ist:	☐ Operation an den Eierstöcken<br>☐ Prüfung der Durchgängigkeit der Eileiter (=Chromopertubation/Blauprobe)<br>☐ bariatrische Operationen (z.B. Magen-Bypass)<br>☐ Andere (wie oben beschrieben)<br>☐ Unbekannt<br>(Falls Sie nicht wissen, was operiert werden soll, kreuzen Sie "Unbekannt" an.)
Wurden Sie von Ihrer Frauenärztin / Ihrem Frauenarzt bei den Therapieversuchen betreut?	◯ Ja<br>◯ Nein
Wie zufrieden sind Sie mit der Betreuung der Therapie durch Ihre Frauenärztin / Ihren Frauenarzt?	0 50 100<br>*(Place a mark on the scale above)*
Hätten Sie sich bei der Therapie eine Betreuung durch Ihre Frauenärztin / Ihren Frauenarzt gewünscht?	◯ Ja<br>◯ Nein

Fruchtbarkeitsgefühl

Fühlen Sie sich in Ihrer Fruchtbarkeit gestört oder eingeschränkt?	◯ Ja<br>◯ Nein
Wurden Sie von Ihrer Frauenärztin / Ihrem Frauenarzt bezüglich dieses gestörten Fruchtbarkeitsgefühls beraten?	◯ Ja<br>◯ Nein
Wie zufrieden sind Sie mit der Beratung bezüglich dieses gestörten Fruchtbarkeitsgefühls?	0 50 100<br>*(Place a mark on the scale above)*

Hätten Sie sich bezüglich dieses gestörten Fruchtbarkeitsgefühl eine Beratung durch Ihre Frauenärztin / Ihren Frauenarzt gewünscht?

- ◯ Ja
- ◯ Nein

Gesamtzufriedenheit Kinderwunsch

Wie zufrieden sind Sie gesamthaft mit der Betreuung durch Ihre Frauenärztin / Ihren Frauenarzt bezüglich Kinderwunsch, Schwangerschaft und Fruchtbarkeit?

0 — 50 — 100

(Place a mark on the scale above)

Würden Sie sich zusätzliche Beratung wünschen?

- ◯ Ja
- ◯ Nein

Bitte kreuzen Sie an, was Sie sich zusätzlich wünschen würden:

- ☐ mehr Beratung / Beruhigung
- ☐ mehr Abgabe von Informationen (z.B. Broschüren)
- ☐ mehr Möglichkeiten Fragen zu stellen
- ☐ mehr Tests (Bluttests, Ultraschall, etc.)
- ☐ mehr Therapie-Möglichkeiten
- ☐ Andere

Welche andere zusätzliche Beratung?

____
(Bitte genauer beschreiben.)

Einflussbereich 5 - Psyche & Emotion

Bitte beantworten Sie die untenstehenden Fragen.

Herzlichen Dank!

Körperbild

Machen Sie sich oft Sorgen darüber wie Sie aussehen und wünschen Sie könnten weniger daran denken?	◯ Ja<br>◯ Nein
Machen Sie sich an einem typischen Tag über eine Stunde Sorgen über Ihr Aussehen?	◯ Ja<br>◯ Nein
Machen Ihre Sorgen es schwierig Ihren Job zu erledigen oder mit Familie und Freunden zusammen zu sein?	◯ Ja<br>◯ Nein

Betrachten Sie bitte die folgenden Figuren und beantworten Sie die folgenden zwei Fragen dazu:

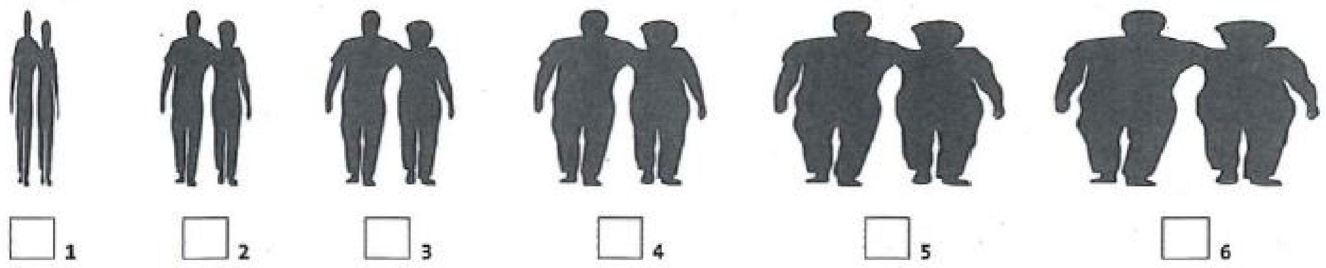

Welches der Bilder zeigt am besten, wie Sie sich selbst sehen?	◯ 1<br>◯ 2<br>◯ 3<br>◯ 4<br>◯ 5<br>◯ 6
Welches der Bilder zeigt am besten, wie Sie gerne wären?	◯ 1<br>◯ 2<br>◯ 3<br>◯ 4<br>◯ 5<br>◯ 6
Wurden Sie von Ihrer Frauenärztin / Ihrem Frauenarzt bezüglich Ihres Körperbildes beraten?	◯ Ja<br>◯ Nein<br>(Wurden Ihnen diese oder ähnliche Fragen gestellt oder wurde über das Thema Körperbild gesprochen?)
Wie zufrieden sind Sie mit der Beratung bezüglich Körperbild?	0 50 100<br>*(Place a mark on the scale above)*
Hätten Sie sich zum Thema Körperbild eine Beratung durch Ihre Frauenärztin / Ihren Frauenarzt gewünscht?	◯ Ja<br>◯ Nein

Essgewohnheiten

Spielt Ihr Gewicht eine Rolle dabei, wie Sie sich fühlen?	◯ Ja<br>◯ Nein
Sind Sie zufrieden mit Ihren Essgewohnheiten?	◯ Ja<br>◯ Nein
Wurden Sie von Ihrer Frauenärztin / Ihrem Frauenarzt bezüglich Essgewohnheiten beraten?	◯ Ja<br>◯ Nein<br>(Wurden Ihnen diese oder ähnliche Fragen gestellt oder wurde über das Thema Essgewohnheiten gesprochen?)
Wie zufrieden sind Sie mit der Beratung bezüglich Essgewohnheiten?	0 50 100<br>*(Place a mark on the scale above)*
Hätten Sie sich zum Thema Essgewohnheiten eine Beratung durch Ihre Frauenärztin / Ihren Frauenarzt gewünscht?	◯ Ja<br>◯ Nein

Angst & Depression bzw. Gefühle

Wie oft fühlten Sie sich in den letzten 2 Wochen durch folgende Beschwerden beeinträchtigt?

Niedergeschlagenheit, Schwermut oder Hoffnungslosigkeit	◯ Ständig (fast jeden Tag)<br>◯ Oft (mehr als Hälfte der Tage)<br>◯ Manchmal (Hälfte der Tage)<br>◯ Selten (einzelne Tage)<br>◯ Nie
Weniger Interesse oder Freude an Ihren Tätigkeiten	◯ Ständig (fast jeden Tag)<br>◯ Oft (mehr als Hälfte der Tage)<br>◯ Manchmal (Hälfte der Tage)<br>◯ Selten (einzelne Tage)<br>◯ Nie
Nervosität, Ängstlichkeit, Anspannung oder übermässiger Sorge	◯ Ständig (fast jeden Tag)<br>◯ Oft (mehr als Hälfte der Tage)<br>◯ Manchmal (Hälfte der Tage)<br>◯ Selten (einzelne Tage)<br>◯ Nie
Nicht in der Lage aufzuhören, sich Sorgen zu machen	◯ Ständig (fast jeden Tag)<br>◯ Oft (mehr als Hälfte der Tage)<br>◯ Manchmal (Hälfte der Tage)<br>◯ Selten (einzelne Tage)<br>◯ Nie
Wurden Sie von Ihrer Frauenärztin / Ihrem Frauenarzt schon einmal bezüglich solcher Gefühle beraten?	◯ Ja<br>◯ Nein
Wie zufrieden sind Sie mit der Beratung bezüglich solcher Gefühle?	0 50 100<br>*(Place a mark on the scale above)*

Hätten Sie sich eine Beratung bezüglich solcher Gefühle durch Ihre Frauenärztin / Ihren Frauenarzt gewünscht?	◯ Ja<br>◯ Nein

Gesamtzufriedenheit psychische & emotionale Gesundheit

Wie zufrieden sind Sie gesamthaft mit der Betreuung durch Ihre Frauenärztin / Ihren Frauenarzt bezüglich Ihrer psychischen und emotionalen Gesundheit?	0 50 100<br>*(Place a mark on the scale above)*
Würden Sie sich zusätzliche Beratung wünschen?	◯ Ja<br>◯ Nein
Bitte kreuzen Sie an, was Sie sich zusätzlich wünschen würden:	☐ mehr Beratung / Beruhigung<br>☐ mehr Abgabe von Informationen (z.B. Broschüren)<br>☐ mehr Möglichkeiten Fragen zu stellen<br>☐ mehr Tests (Bluttests, Ultraschall, etc.)<br>☐ mehr Therapie-Möglichkeiten<br>☐ Andere
Welche andere zusätzliche Beratung?	____________________<br>(Bitte genauer beschreiben.)

Risikomonitoring

Bitte beantworten Sie die untenstehenden Fragen.

Herzlichen Dank!

Hat Ihre Frauenärztin / Ihr Frauenarzt mit Ihnen über Risiken gesprochen, die bei PCOS erhöht sein können?	◯ Ja<br>◯ Nein<br>(Falls Sie nicht sicher sind, was damit gemeint ist, können Sie "Ja" anklicken und die Unterkategorien anschauen. Falls nichts Zutreffendes dabei ist, können Sie auch wieder auf "Nein" wechseln.)
Bitte kreuzen Sie an, welche Themen angesprochen wurden:	☐ Herz-Kreislauf-Erkrankungen (z.B. Bluthochdruck, Herzinfarkt, Schlaganfall, Thrombose, Lungenembolie, etc.)<br>☐ Zucker-Erkrankungen (z.B. Diabetes, Diabetes-Vorstufen, Schwangerschaftsdiabetes)<br>☐ Obstruktive Schlaf-Apnoe (Atemaussetzer beim Schlafen)<br>☐ Endometrium-Krebs (Krebs der Gebärmutter-Schleimhaut)<br>☐ Andere
Welche anderen Risiken?	______________________________<br>(Bitte genauer beschreiben.)

Herzkreislauf-Erkrankungen und Gewicht

Bitte kreuzen Sie an was bei Ihnen zutrifft oder bekannt ist:	☐ Raucherin<br>☐ Übergewicht<br>☐ hohe Cholesterinwerte<br>☐ Bluthochdruck<br>☐ gestörte Zucker-Toleranz oder Diabetes<br>☐ Bewegungsmangel<br>☐ Herz-Kreislauf-Erkrankungen in der Familie (z.B. Herzinfarkt, Schlaganfall)<br>☐ Keines der genannten
Haben/hatten Sie schon eine Herzkreislauf-Erkrankung?	◯ Ja<br>◯ Nein<br>(Falls Sie nicht sicher sind, was damit gemeint ist, können Sie "Ja" anklicken und die Unterkategorien anschauen. Falls nichts Zutreffendes dabei ist, können Sie auch wieder auf "Nein" wechseln.)
Bitte kreuzen Sie die Zutreffenden an:	☐ Bluthochdruck<br>☐ Herzinfarkt<br>☐ Schlaganfall<br>☐ Lungenembolie<br>☐ Thrombose / Gefässverschluss (z.B. im Bein/Arm)<br>☐ Andere

Welche anderen Herzkreislauf-Erkrankungen?	__________________________________ (Bitte genauer beschreiben.)
Wird Ihr Gewicht bei Ihrer Frauenärztin / Ihrem Frauenarzt regelmässig gemessen?	◯ Ja<br>◯ Nein<br>(Regelmässig bedeutet mindestens einmal pro Jahr.)
Wird Ihr Blutdruck bei Ihrer Frauenärztin / Ihrem Frauenarzt regelmässig gemessen?	◯ Ja<br>◯ Nein<br>(Regelmässig bedeutet mindestens einmal pro Jahr.)
Wird Ihr Cholesterin-Wert im Blut bei Ihrer Frauenärztin / Ihrem Frauenarzt regelmässig gemessen?	◯ Ja<br>◯ Nein<br>◯ Unbekannt<br>(Regelmässig bedeutet mindestens einmal pro Jahr.)
Wird Ihr Cholesterin-Wert im Blut bei Ihrer Hausärztin / Ihrem Hausarzt regelmässig gemessen?	◯ Ja<br>◯ Nein<br>◯ Unbekannt<br>(Regelmässig bedeutet mindestens einmal pro Jahr.)
Wurden Sie von Ihrer Frauenärztin / Ihrem Frauenarzt bezüglich erhöhtem Risiko für Herzkreislauf-Erkrankungen bei PCOS und den Risikofaktoren für Herzkreislauf-Erkrankungen beraten?	◯ Ja<br>◯ Nein
Wie zufrieden sind Sie mit der Beratung bezüglich Risiko für Herzkreislauf-Erkrankungen?	0 50 100<br>*(Place a mark on the scale above)*
Hätten Sie sich dazu eine Beratung durch Ihre Frauenärztin / Ihren Frauenarzt gewünscht?	◯ Ja<br>◯ Nein

Zucker-Toleranz und Diabetes

Wird Ihr Blutzuckerwert bei Ihrer Frauenärztin / Ihrem Frauenarzt regelmässig gemessen?	◯ Ja<br>◯ Nein<br>◯ Unbekannt<br>(Regelmässig bedeutet hier mindestens einmal alle drei Jahre.)
Wird Ihr Blutzuckerwert bei Ihrer Hausärztin / Ihrem Hausarzt oder Ihrem Diabetes-Spezialisten regelmässig gemessen?	◯ Ja<br>◯ Nein<br>◯ Unbekannt<br>(Regelmässig bedeutet hier mindestens einmal alle drei Jahre.)
Wurde bei Ihnen vor der Schwangerschaft ein Zuckerbelastungstest (=oGTT) durchgeführt?	◯ Ja<br>◯ Nein<br>◯ Unbekannt<br>(nüchtern Blutzuckermessung, dann Zuckerlösung trinken, nach 1 Stunde erneute Messung)

Wurde bei Ihnen während der Schwangerschaft ein Zuckerbelastungstest (=oGTT) durchgeführt?	◯ Ja<br>◯ Nein<br>◯ Unbekannt<br>(nüchtern Blutzuckermessung, dann Zuckerlösung trinken, nach 1 Stunde erneute Messung)
Wurden Sie von Ihrer Frauenärztin / Ihrem Frauenarzt bezüglich erhöhtem Risiko für Zuckerkrankheiten bei PCOS beraten?	◯ Ja<br>◯ Nein
Wie zufrieden sind Sie mit der Beratung bezüglich Risiko für Zuckerkrankheiten?	0 50 100<br>*(Place a mark on the scale above)*
Hätten Sie sich bezüglich Risiko für Zuckererkrankungen eine Beratung durch Ihre Frauenärztin / Ihren Frauenarzt gewünscht?	◯ Ja<br>◯ Nein

Obstruktive Schlafapnoe

Bitte kreuzen Sie an was bei Ihnen zutrifft oder bekannt ist:	☐ Schnarchen<br>☐ Atemaussetzer im Schlaf (z.B. von Partner beobachtet)<br>☐ unerholtes Aufwachen<br>☐ Tagesschläfrigkeit<br>☐ morgendliche Kopfschmerzen<br>☐ Konzentrationsstörungen<br>☐ Keines der genannten
Ist bei Ihnen ein Schlafapnoe-Syndrom bekannt?	◯ Ja<br>◯ Nein<br>(Nur "Ja" ankreuzen, wenn dieses von einem Arzt festgestellt wurde.)
Waren Sie schon einmal bei einem Spezialisten für Schlafmedizin oder in einem Schlaflabor?	◯ Ja<br>◯ Nein
Wurden Sie von Ihrer Frauenärztin / Ihrem Frauenarzt bezüglich erhöhtem Risiko für Schlafapnoe (nächtliche Atemaussetzer) beraten?	◯ Ja<br>◯ Nein
Wie zufrieden sind Sie mit der Beratung bezüglich Risiko für Schlafapnoe?	0 50 100<br>*(Place a mark on the scale above)*
Hätten Sie sich bezüglich Risiko für Schlafapnoe eine Beratung durch Ihre Frauenärztin / Ihren Frauenarzt gewünscht?	◯ Ja<br>◯ Nein

Endometrium-Krebs

Leiden oder litten Sie an Endometrium-Krebs?	◯ Ja<br>◯ Nein<br>(Endometrium-Krebs = Krebs der Gebärmutterschleimhaut)
Wurden Sie von Ihrer Frauenärztin / Ihrem Frauenarzt bezüglich leicht erhöhtem Risiko für Gebärmutter-Krebs beraten?	◯ Ja<br>◯ Nein
Wie zufrieden sind Sie mit der Beratung bezüglich leicht erhöhtem Risiko für Gebärmutterkrebs?	0 50 100<br>*(Place a mark on the scale above)*
Hätten Sie sich eine Beratung bezüglich leicht erhöhtem Risiko für Gebärmutterkrebs gewünscht?	◯ Ja<br>◯ Nein

Gesamtzufriedenheit Risikomonitoring

Wie zufrieden sind Sie gesamthaft mit der Betreuung durch Ihre Frauenärztin / Ihren Frauenarzt bezüglich Risiko für andere Erkrankungen?	0 50 100<br>*(Place a mark on the scale above)*
Würden Sie sich zusätzliche Beratung wünschen?	◯ Ja<br>◯ Nein
Bitte kreuzen Sie an, was Sie sich zusätzlich wünschen würden:	☐ mehr Beratung / Beruhigung<br>☐ mehr Abgabe von Informationen (z.B. Broschüren)<br>☐ mehr Möglichkeiten Fragen zu stellen<br>☐ mehr Tests (Bluttests, Gewicht, Blutdruck, etc.)<br>☐ mehr Therapie-Möglichkeiten<br>☐ Andere
Welche andere zusätzliche Beratung?	____________________<br>(Bitte genauer beschreiben.)

Schlussfragen

Bitte beantworten Sie die untenstehenden Fragen.

Herzlichen Dank!

Wenn Sie nun alle Bereiche, in denen Sie von Ihrem PCOS betroffen sind, gesamthaft betrachten: Wie zufrieden sind Sie grundsätzlich mit der gesamten Beratung bezüglich PCOS durch Ihre Frauenärztin / Ihren Frauenarzt?

0 50 100

(Place a mark on the scale above)

Würden Sie sich gesamthaft zusätzliche Beratung wünschen?

- ◯ Ja
- ◯ Nein

Bitte kreuzen Sie an, was Sie sich gesamthaft zusätzlich wünschen würden:

- ☐ mehr Beratung / Beruhigung
- ☐ mehr Abgabe von Informationen (z.B. Broschüren)
- ☐ mehr Möglichkeiten Fragen zu stellen
- ☐ mehr Tests (Bluttests, Ultraschall, etc.)
- ☐ mehr Therapie-Möglichkeiten
- ☐ Andere

Welche andere zusätzliche Beratung?

____

(Bitte genauer beschreiben.)

Hat diese Umfrage etwas an Ihrer Zufriedenheit mit Ihrer Frauenärztin / Ihrem Frauenarzt geändert?

- ◯ Ja
- ◯ Nein

Wie gut betreut haben Sie sich vorher gefühlt?

0 50 100

(Place a mark on the scale above)

Inwiefern hat die Umfrage Ihre Einstellung verändert?

____

(Bitte genauer beschreiben.)

Wir möchten Sie darauf hinweisen, dass diese Umfrage alle möglichen Betreuungs-Punkte gemäss neuer Leitlinien abdeckt. Falls Ihre Frauenärztin / Ihr Frauenarzt nicht alles entsprechend gleich macht, heisst dies nicht, dass sie/er es schlecht macht. Falls Sie nach dieser Umfrage Unsicherheiten haben oder sich schlechter betreut fühlen, melden Sie sich bitte bei Ihrer Frauenärztin / Ihrem Frauenarzt und besprechen Sie die Situation mit ihr/ihm. Dafür gibt es sicher eine Lösung.

Hat Ihnen in dieser Umfrage eine Frage gefehlt, die Ihnen wichtig erscheint?

- ◯ Ja
- ◯ Nein

Bitte schreiben Sie die fehlende/n Frage/n auf:

____

Möchten Sie sonst noch etwas sagen?

- ◯ Ja
- ◯ Nein

Kommentar:	__________________________________
Wie sind Sie auf die Umfrage gestossen?	☐ Frauenärztin/-arzt in Praxis<br>☐ Frauenärztin/-arzt im Spital<br>☐ Plakat / Flyer<br>☐ Social Media<br>☐ Universität<br>☐ Internet-Forum<br>☐ Selbsthilfe-Gruppe<br>☐ Freunde / Bekannte<br>☐ Andere
Über welche andere Möglichkeit?	__________________________________
Über welchen Social-Media Kanal?	☐ Facebook<br>☐ Instagram<br>☐ LinkedIn<br>☐ Andere
Über welchen anderen Social-Media Kanal?	__________________________________
