# Supplementary material for: Unmet clinical needs in women with polycystic ovary syndrome regarding fertility and obesity: a cross-sectional study from the patient’s perspective
Source: Arch Gynecol Obstet. 2025 Jan 25;311(3):851–9. doi: 10.1007/s00404-024-07916-1 (PMC11920305; doi:10.1007/s00404-024-07916-1)
Supplement: Supplementary file 3 — Supplementary file3 (PDF 665 KB) [file 404_2024_7916_MOESM3_ESM.pdf]

File S3: Ethical approvals

Kanton Bern
Canton de Berne

Gesundheits-, Sozial- und Integrationsdirektion
Kantonale Ethikkommission für die Forschung

Murtenstrasse 31
3010 Bern
Bern
+41 31 633 70 70 (Telefon)
+41 31 633 70 71 (Telefax)
info.kek.kapa@be.ch
www.be.ch/gsi

Dorothy Pfiffner
+41 31 633 70 77
dorothy.pfiffner@be.ch

GSI-KEK, Murtenstrasse 31, 3010 Bern

Julia Estermann
Muriweid 4
6207 Nottwil

Zuständigkeitsabklärung

BASEC-Nr: Req-2020-00801 **Eingangsdatum:** 02/07/2020

Titel: Versorgungssituation und -bedarf bei Frauen mit PCOS

Ergebnis der Zuständigkeitsabklärung

☒ **Nicht zuständig**, d.h. das Vorhaben ist nicht bewilligungspflichtig
Begründung: Das Vorhaben fällt nicht unter das Humanforschungsgesetz, Art. 2, Abs. 1

☐ **Zuständig**: Bewilligung gemäss Humanforschungsgesetz, Art. 2, Abs. 1 **notwendig**.
Bitte reichen Sie der KEK ein Gesuch gemäss www.swissethics.ch ein

Gebühren: CHF 200.-- (Tarifcode 6.0)
Rechnung folgt

Datum/Ort: 08.07.2020/Bern

i.V.

Prof. Dr. med. Christian Seiler
Präsident

Dr. sc. nat. Dorothy Pfiffner
Leiterin wissenschaftliches Sekretariat

FACHBEREICH MEDIZIN FRANKFURT
GOETHE-UNIVERSITÄT

Ethikkommission des Fachbereichs Medizin der Goethe-Universität
c/o Universitätsklinikum ·Theodor-Stern-Kai 7 ·60590 Frankfurt am Main

IM HAUSE
Klinik für Frauenheilkunde und Geburtshilfe
Endokrinologie und Reproduktionsmedizin
Frau Dr. Annette Bachmann

KFG

Ethik-Kommission

Vorsitz:
Prof. Dr. Sebastian Harder

Geschäftsführung:
Dr. Johannes Hätscher

Bearbeitung des Vorgangs:
Klinge

Dienstag, 08. Dezember 2020

Titel: Versorgungssituation und –bedarf bei Frauen mit PCOS

Sehr geehrte Frau Dr. Bachmann,

vielen Dank für die Zusendung der Studienunterlagen vom 23.11.2020.

Für die o.g. anonymisierte Datenerhebung besteht keine Pflicht zur berufsrechtlichen Beratung durch die Ethikkommission des Fachbereiches Medizin der Goethe Universität.

Mit freundlichen Grüßen

S. Harder

Prof. Dr. med. Sebastian Harder
Vorsitzender der Ethik-Kommission

Vorgelegte Unterlagen:
- Fragenkatalog Version 5

Geschäftsstelle

Sekretariat:
Ruth Schmidt
Tel.: 069 / 6301-3758
Fax: 069 / 6301-83434
E-Mail: ethikkommission@kgu.de

Mitarbeiter/innen:
Durchwahl
Dorothea Bittner Tel.: 3889
Angela Heuser Tel.: 4552
Ina Klinge Tel.: 3884
Dr. Marnie Kopp Tel.: 3884
Myriam Freund Tel.: 7239
Fax: 83434
E-Mail: ethikkommission@kgu.de

http://www.kgu.de/ueber-uns/vorstand-des-universitaetsklinikums/dekan/home/

Lieferadresse:
Ethik-Kommission des
Fachbereichs Medizin
der Goethe-Universität
c/o Universitätsklinikum
Theodor-Stern-Kai 7
Haus 1, 2. OG, Zi. 207
60590 Frankfurt am Main

Öffnungszeiten f. Anlieferungen:
Montag bis Donnerstag:
09:00 bis 15:30 Uhr
Freitag:
09:00 bis 12:30 Uhr
