# Supplementary material for: Unmet clinical needs in women with polycystic ovary syndrome regarding fertility and obesity: a cross-sectional study from the patient’s perspective
Source: Arch Gynecol Obstet. 2025 Jan 25;311(3):851–9. doi: 10.1007/s00404-024-07916-1 (PMC11920305; doi:10.1007/s00404-024-07916-1)
Supplement: Supplementary file 4 — Supplementary file4 (PDF 111 KB) [file 404_2024_7916_MOESM4_ESM.pdf]

Supplementary Table S1: Starting variables included in the stepwise backwards multivariate regression model in Table 6 (Variable/Field Name according to Suppl. File S1).

age	kw_ss_infert_th2_spec___1
bmi	kw_ss_infert_th2_spec___2
kw_act_nr2	kw_ss_infert_th2_spec___3
mens_past_regular_yn	kw_ss_infert_th2_spec___4
mens_current_regular_yn	kw_ss_infert_th2_spec___88
kw_ss_yn	kw_ss_infert_th2_spec___99
employment___1	kw_ss_infert_th3_spec___1
employment___2	kw_ss_infert_th3_spec___2
employment___3	kw_ss_infert_th3_spec___3
employment___4	kw_ss_infert_th_act_spec___1
employment___5	kw_ss_infert_th_act_spec___2
employment___6	kw_ss_infert_th_act_spec___3
employment___7	kw_ss_infert_th_act_spec___4
risk_cvrf___1	kw_ss_infert_th_act_spec___88
risk_cvrf___2	kw_ss_infert_th1_act_spec___1
risk_cvrf___3	kw_ss_infert_th1_act_spec___2
risk_cvrf___4	kw_ss_infert_th1_act_spec___3
risk_cvrf___5	kw_ss_infert_th1_act_spec___4
risk_cvrf___6	kw_ss_infert_th1_act_spec___5
risk_cvrf___7	kw_ss_infert_th1_act_spec___6
risk_cvrf___0	kw_ss_infert_th1_act_spec___7
risk_cvd_yn	kw_ss_infert_th1_act_spec___8
kw_ss_infert_th_spec___1	kw_ss_infert_th1_act_spec___9
kw_ss_infert_th_spec___2	kw_ss_infert_th1_act_spec___10
kw_ss_infert_th_spec___3	kw_ss_infert_th1_act_spec___88
kw_ss_infert_th_spec___4	kw_addit_yn
kw_ss_infert_th_spec___88	kw_addit_spec___1
kw_ss_infert_th1_spec___1	kw_addit_spec___2
kw_ss_infert_th1_spec___2	kw_addit_spec___3
kw_ss_infert_th1_spec___3	kw_addit_spec___4
kw_ss_infert_th1_spec___4	kw_addit_spec___5
kw_ss_infert_th1_spec___5	kw_addit_spec___88
kw_ss_infert_th1_spec___6	
kw_ss_infert_th1_spec___7	
kw_ss_infert_th1_spec___8	
kw_ss_infert_th1_spec___9	
kw_ss_infert_th1_spec___10	
kw_ss_infert_th1_spec___88	
